# Akebia Saponin D Targeting Ubiquitin Carboxyl‐Terminal Hydrolase 4 Promotes Peroxisome Proliferator‐Activated Receptor Gamma Deubiquitination and Activation of Brown Adipose Tissue Thermogenesis in Obesity

**DOI:** 10.1002/mco2.70420

**Published:** 2025-10-15

**Authors:** Lang Chen, Dong‐Hai Liu, Yu‐Xi Li, Song Yang, Wei‐Hua Jia, Liang Peng, Hong‐Lin Liu, Xing‐Bo Wang, Bing Hu, Yu‐Chen Wang, Calvin Pan, Aldons Jake Lusis, Li‐Hong Liu, Li‐Li Gong

**Affiliations:** ^1^ Institute of Clinical Medical Sciences China‐Japan Friendship Hospital Capital Medical University Beijing China; ^2^ China‐Japan Friendship Hospital (Institute of Clinical Medical Sciences) Chinese Academy of Medical Sciences & Peking Union Medical College Beijing China; ^3^ Beijing Advanced Innovation Center for Soft Matter Science and Engineering College of Life Science and Technology Beijing University of Chemical Technology Beijing China; ^4^ China‐Japan Friendship Hospital Beijing China; ^5^ Department of Pharmacy China‐Japan Friendship Hospital Beijing China; ^6^ Division of Cardiology David Geffen School of Medicine at UCLA Los Angeles California USA

**Keywords:** Akebia Saponin D, deubiquitination, mitochondria, obesity, ubiquitin carboxyl‐terminal hydrolase 4

## Abstract

Promoting thermogenesis in adipose tissue to enhance energy expenditure is widely regarded as a promising strategy for obesity treatment. However, the development of effective thermogenic drugs remains challenging. Our screenings identified the natural compound Akebia Saponin D (ASD) as a potent brown fat thermogenesis activator in mice, showing effects through mitochondrial brown fat uncoupling protein 1 (UCP1)‐dependent pathways. ASD was found to significantly mitigate high‐fat diet‐induced obesity and enhance the mitochondrial quality of brown adipocytes to promote thermogenesis. Utilizing human protein microarrays, cellular thermal shift assay, and drug affinity responsive target stability, along with microscale thermophoresis and molecular docking analysis, we identified ubiquitin carboxyl‐terminal hydrolase 4 (USP4) as a direct target of ASD. ASD interacts with USP4 and promotes the deubiquitination of peroxisome proliferator‐activated receptor gamma, thus inhibiting its proteasomal degradation and enhancing the transcriptional activation of UCP1 in brown adipocytes. Additionally, USP4 knockdown was shown to attenuate brown fat thermogenesis induced by ASD. In summary, our findings demonstrate that ASD promotes brown fat thermogenesis by targeting USP4, highlighting its potential as a promising natural small molecule for obesity treatment.

## Graphical abstract

ASD, a natural plant product screened from small molecule compound library, is an antiobesity agent in mice. ASD promotes BAT thermogenesis to inhibit body weight gain. USP4 was identified and validated as a direct molecular target of ASD. ASD‐induced thermogenesis is modulated by a USP4–PPARγ–UCP1 axis.

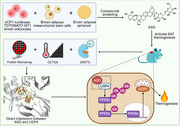

## 1 Introduction

Obesity refers to the pathological buildup of lipids resulting from an imbalance between caloric intake and expenditure over an extended period. This condition is often concomitant with various comorbidities and metabolic disorders [1]. Over the past three decades, the global prevalence of obesity has more than doubled, making its treatment a critical public health priority [2]. Thermogenesis in adipose tissue can regulate energy metabolism, offering a potential therapeutic strategy for obesity [3, 4]. The classic thermogenesis is the futile cycle mediated by uncoupling protein 1 (UCP1), which facilitates the release of energy produced by mitochondria as heat [5, 6, 7]. Thermogenic brown adipose tissue (BAT) plays a pivotal role in the regulation of body temperature in response to cold, primarily through UCP1‐dependent mechanisms [8, 9].

Adult individuals possess functional BAT that is capable of contributing to thermogenesis; however, its activity is diminished in individuals with obesity [10, 11, 12, 13]. In response to cold exposure or certain dietary stimuli, the sympathetic nervous system releases norepinephrine, which activates β‐3‐adrenergic receptors (β3‐AR) in adipose tissue [14]. This activation triggers the cyclic adenosine monophosphate signaling pathway, leading to the nuclear activation of transcription factors such as cyclic AMP‐responsive element‐binding protein 1, histone‐lysine N‐methyltransferase PRDM16, cyclic AMP‐dependent transcription factor ATF‐2, and peroxisome proliferator‐activated receptor gamma (PPARγ), which in turn promote UCP1 transcription [15, 16, 17, 18]. Stimulating the browning of white fat and activating BAT thermogenesis are considered potential treatments and interventions for obesity. However, most of the β3‐AR agonists have been found to have side effects, such as cardiovascular risks, due to off‐target effects of β3‐AR on other tissues [1, 19]. In addition, studies have demonstrated that the activation of thermogenesis signals in human brown adipocytes is dependent on β‐2‐adrenergic receptors [20]. Consequently, developing antiobesity drugs that promote adipocyte browning and activate BAT thermogenesis remains a significant challenge.

PPARγ, a member of the nuclear receptor superfamily of ligand‐induced transcription factors, is predominantly and highly expressed in adipose tissue [21]. PPARγ can enhance the expression of particular genes within brown adipocytes, inhibit the expression of certain genes in white adipocytes, and induce the appearance of beige phenotype in white adipocytes [22]. Previous studies have shown that various posttranslational modifications, including deacetylation, phosphorylation, sumoylation, and ubiquitination, are involved in the regulation of PPARγ activity [23, 24, 25].

Ubiquitination refers to the process in which ubiquitin (Ub) molecules are covalently attached to lysine residues of target proteins through the coordinated action of E1 Ub‐activating enzyme, E2 Ub‐conjugating enzyme, and E3 Ub ligase. This process facilitates the synthesis of Ub chains on target proteins, marking them for recognition and degradation by the proteasome [26, 27]. Deubiquitinating enzymes (DUBs) remove the ubiquitination of proteins by reversing the synthesis of Ub chains [28]. Ub carboxyl‐terminal hydrolase 4 (USP4), also known as ubiquitous nuclear protein, was initially identified by Gupta et al. [29] as a mouse gene associated with the tre oncogene. Previous studies have found that USP4 plays a key inhibitory role in nonalcoholic fatty liver disease (NAFLD) and related metabolic disorders [30].

We identified a natural small molecule compound Akebia Saponin D (ASD), which targets USP4. ASD is a triterpenoid saponin derived from traditional Chinese herbal medicine, and can markedly enhance thermogenesis in brown adipocytes. In vivo, ASD targets USP4 to promote the deubiquitination of PPARγ, activate *Ucp1* transcription, and mediate BAT thermogenesis. This intervention leads to a marked reduction in weight gain and notable alleviation of obesity in mice that are fed a high‐fat diet (HFD). Our research aims to explore the effect and mechanism of ASD in alleviating the occurrence and development of obesity. This study provides new ideas, drugs, and targets for obesity prevention and control, laying the foundation for the development of innovative antiobesity drugs.

## 2 Results

### 2.1 Small Molecule Compound Library Screening Brown Fat Thermogenic Agonists

**FIGURE 1:**
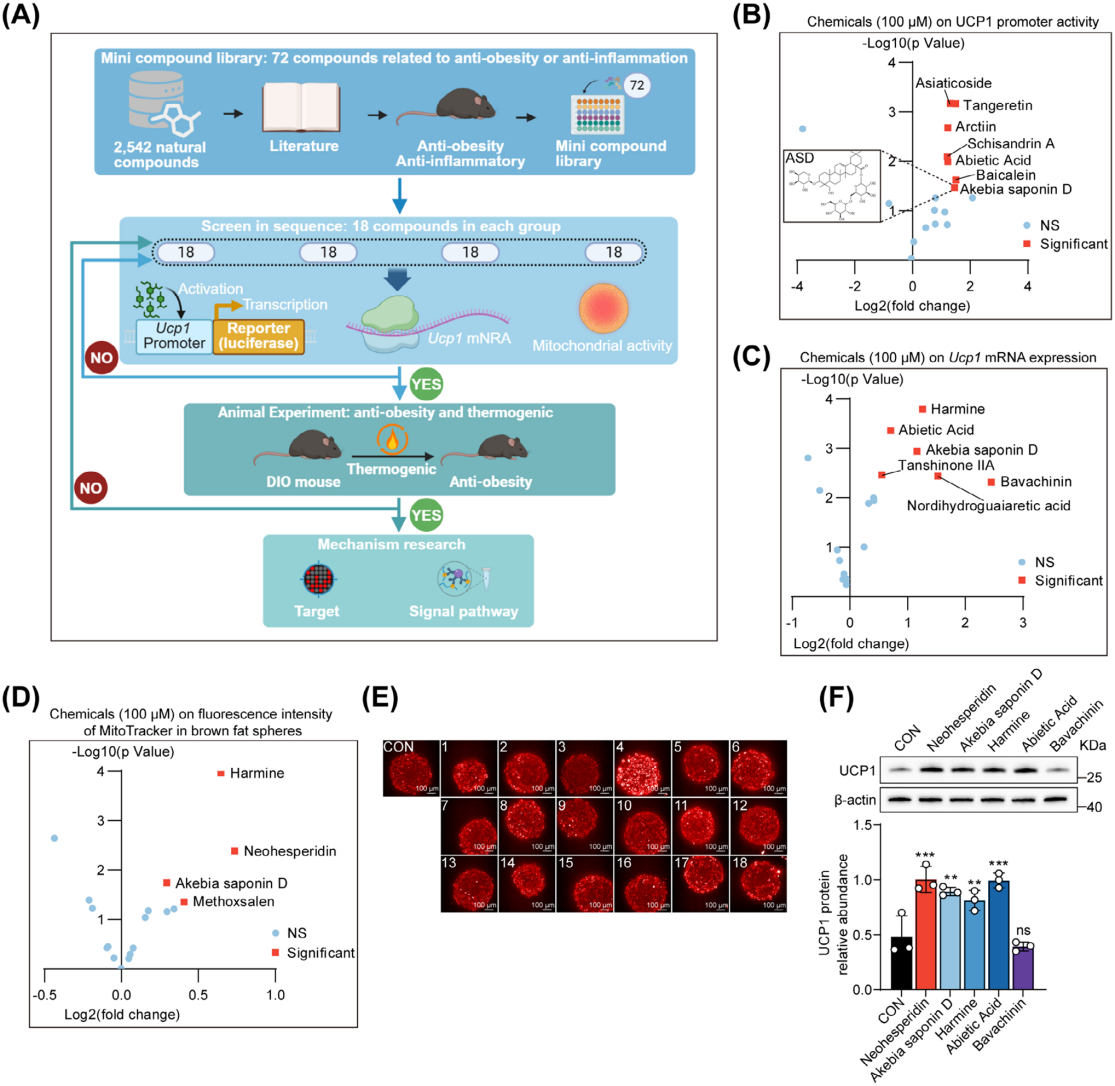
Small molecule compound library screening brown fat thermogenic agonists. (A) Schematic diagram of the screening process for natural small molecule compounds (created with BioRender.com). (B) *Ucp1* promoter activity of brown adipocytes treated with 18 different chemical compounds at the dose of 100 µM for 24 h (*n* = 3). (C) Ucp1 mRNA levels of brown adipocytes treated with compounds at 100 µM for 24 h (*n* = 3). (D) The isolated primary adipose precells were induced into globular‐like cells and differentiated into mature adipocytes, which were treated with drugs for 24 h (*n* = 4). (E) High‐content imaging scan images. See Table S2 for the name of the compound corresponding to the number. Scale bar, 100 µm. (F) UCP1 protein concentration in brown adipocytes treated with compounds at 100 µM for 48 h (*n* = 3). Statistical significance was defined as *p < 0.05, **p < 0.01, and ***p < 0.001. ns, not significant.

To screen out the natural active compounds that can activate the thermogenic effect of BAT, we selected 72 commercially available natural compounds related to antiobesity or anti‐inflammation from a natural product library containing 2542 compounds (Table S1). We divided these 72 compounds into groups of 18 and conducted screening experiments until the most stable compound was identified for subsequent mouse experiments (Figure 1A). These compounds were tested using UCP1 luciferase reporter gene assays in brown adipocytes to assess their ability to stimulate UCP1 expression. Two‐dimensional plots were generated based on fold change (FC) and significance (Figure 1B). Based on this, we further evaluated the mRNA abundance of *Ucp1* in brown adipocytes (Figure 1C). Brown fat organoids were generated and used to assess the effects of these compounds on mitochondrial function (Figures 1D,E and S1A). The red dots in the first quadrant signify compounds potentially capable of stimulating BAT (*p* < 0.05, FC > 1.2). We selected five small molecules that exhibited the largest FC in a single experiment or were significantly altered in at least two experiments, which may be associated with activated BAT thermogenesis (Table S2). These compounds were administered to brown adipocytes to evaluate their impact on UCP1 protein abundance (Figure 1F). Among them, ASD exhibited the most stable effect. Furthermore, no prior studies have reported on the regulation of adipose tissue thermogenesis by ASD. Therefore, we chose ASD as a suitable compound to further investigate its potential ability to induce brown adipocytes thermogenesis and antiobesity.

### 2.2 ASD Alleviates Obesity in Mice Induced by a HFD

**FIGURE 2:**
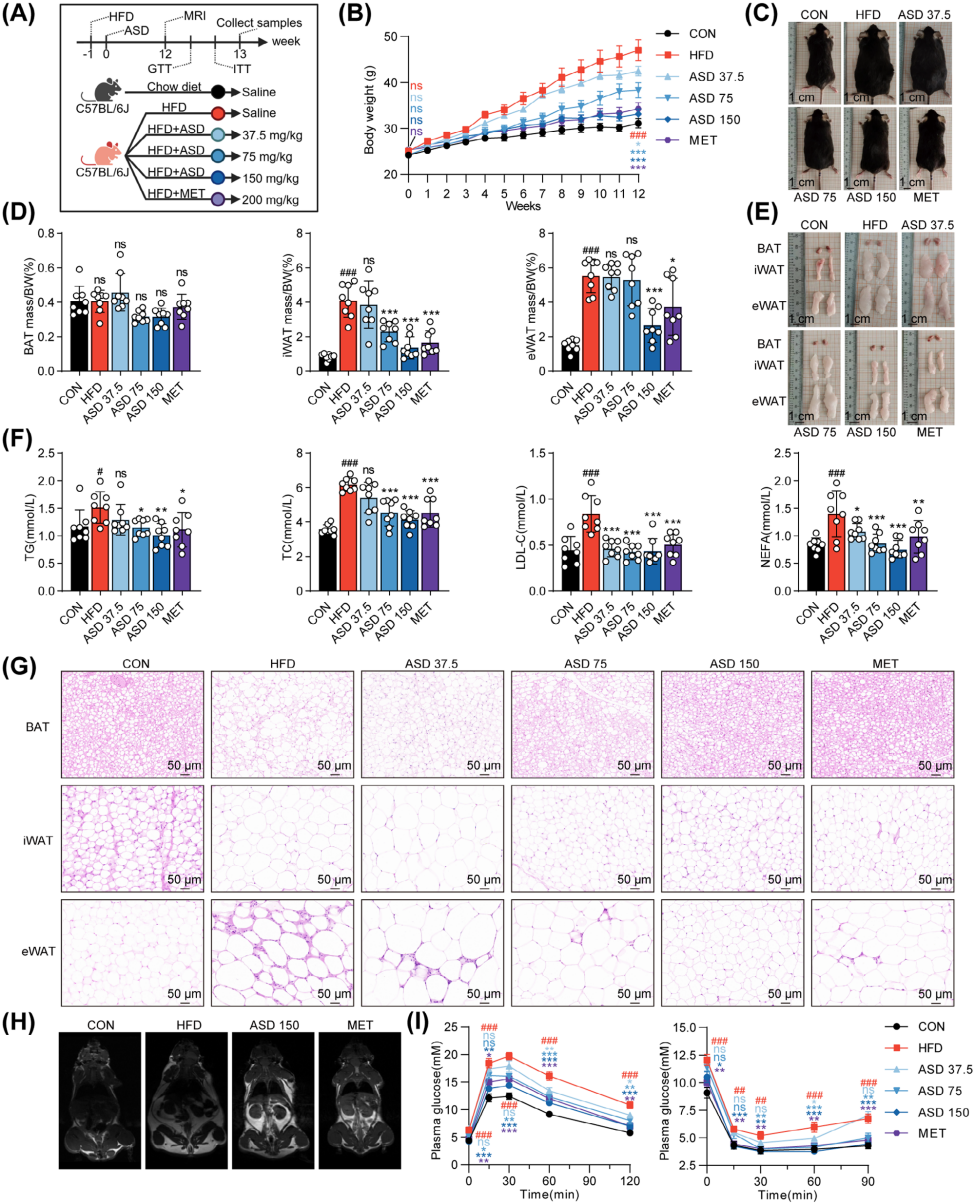
ASD treats obesity in mice caused by a high‐fat diet. (A) The chart shows the treatment of C57BL/6 mice on a high‐fat diet with ASD or MET (created with BioRender.com). (B) Weight changes of C57BL/6 mice during ASD treatment (*n* = 8). (C) Representative photo of a C57BL/6 mouse after ASD treatment. (D) Weights of fat tissue mass normalized by body weight (BW) (*n* = 8). (E) Representative photos of fat pad morphology. (F) Fasting lipid (*n* = 8). (G) Representative H&E staining of dissected tissues. Scale bar, 50 µm. (H) Representative MRI images (*n* = 3). (I) GTT and ITT of DIO mice (*n* = 8). Statistical significance was defined as *p < 0.05, **p < 0.01, and ***p < 0.001. The significance of the control group versus the model group was expressed as #*p* < 0.05, ##*p* < 0.01, and ###*p* < 0.001. ns, not significant. Significance markers match group colors in the line chart.

We used diet‐induced obese mice (DIO mice) to demonstrate whether ASD has an antiobesity effect. ASD showed a dose‐dependent reduction in weight gain in C57BL/6 mice, with the effect in the high‐dose group comparable to that observed in the metformin (MET) group (Figure 2A–C). Additionally, ASD treatment led to a reduction in the coefficients and volume of inguinal white adipose tissue (iWAT) and epididymal white adipose tissue (eWAT). Although there were no substantial alterations in BAT size or weight, its tissue color exhibited a more pronounced reddish hue following ASD treatment (Figure 2D,E).

Blood biochemical analyses revealed that ASD significantly reduced fasting blood glucose (GLU) and lipid levels, particularly total cholesterol, triglycerides (TGs), low‐density lipoprotein cholesterol, and nonesterified fatty acids. Moreover, ASD substantially decreased levels of aspartate aminotransferase and alanine aminotransferase, suggesting a protective effect on liver function. However, no significant changes were observed in high‐density lipoprotein cholesterol levels (Figures 2F and S2A).

Histological analysis showed a reduction in the size of lipid droplets within adipocytes and decreased inflammatory cell infiltration after ASD treatment (Figures 2G and S2B). To assess the contribution of fat mass and lean mass to weight loss, we conducted small animal MRI scans to measure total body fat in mice. The results indicate that ASD leads to a reduction in fat mass, while having no impact on lean mass (Figures 2H and S2C).

Obesity and insulin resistance have a mutually causal relationship. In order to investigate this, the glucose tolerance test (GTT) and insulin tolerance test (ITT) were conducted. Our findings indicate that mice with ASD exhibited a dose‐dependent improvement in glucose tolerance and insulin sensitivity (Figures 2I and S2D). In addition to attenuating pancreatic insulin secretion, the treatment for ASD also exhibited a decrease in fasting insulin levels and a notable reduction in the insulin resistance index (Figure S2F,E). The findings of this study indicate that ASD effectively mitigates both obesity and insulin resistance.

### 2.3 ASD Increases Mitochondrial Mass in BAT and Promotes Thermogenesis, Rather Than Through Exercise

**FIGURE 3:**
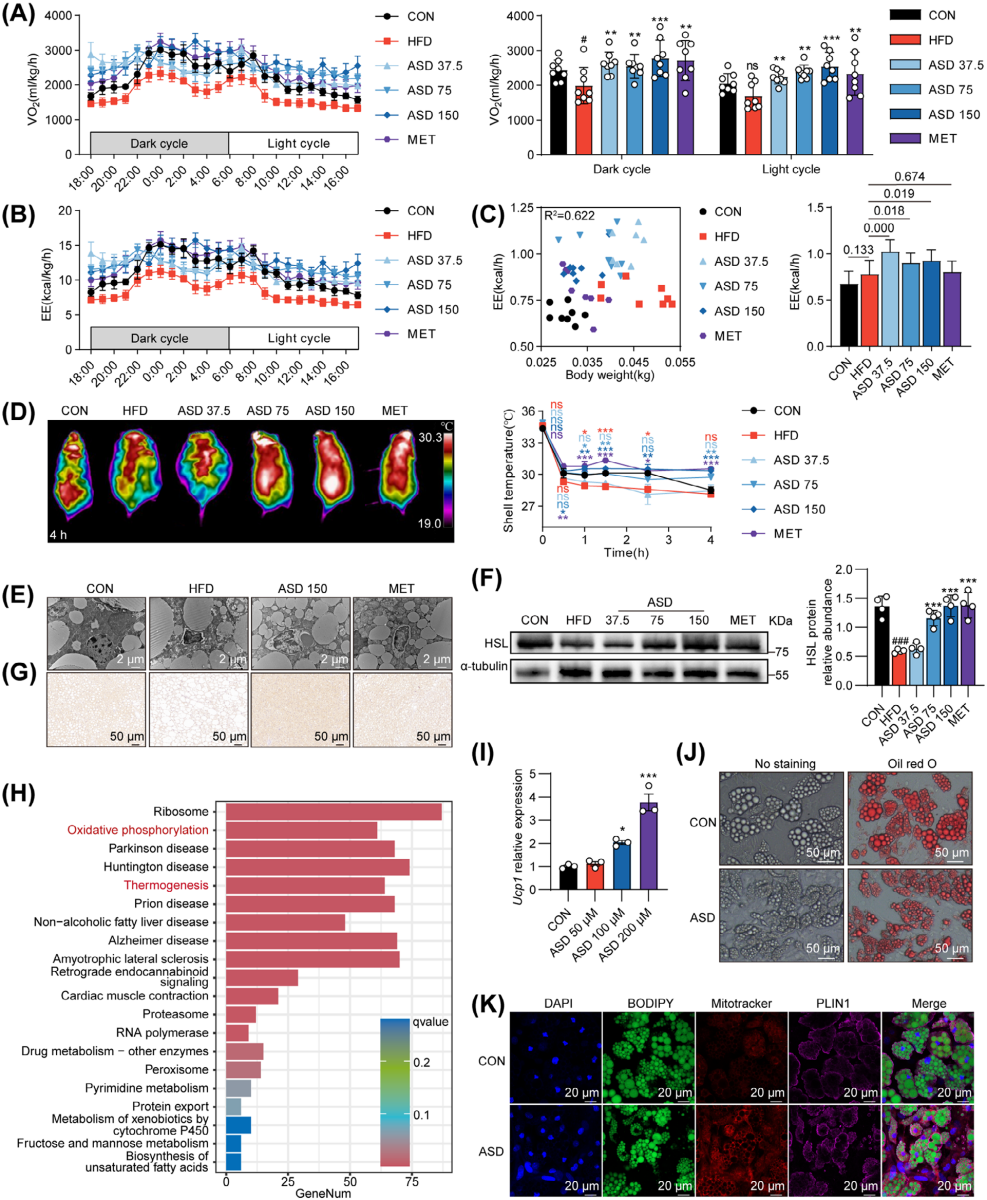
ASD increases mitochondrial mass in BAT and promotes thermogenesis, rather than through exercise. (A) Whole‐body VO_2_ normalized by body weight under basal conditions in DIO mice (*n* = 8). (B) Whole‐body energy exchange normalized by body weight under basal conditions in DIO mice (*n* = 8). (C) Mouse energy expenditure (kJ/h) plotted against body weight (kg), and the adjusted *R* ^2^ = 0.622 (left). Adjusted means of energy expenditure for experimental groups (*n* = 8), analyzed using ANCOVA with body weight as covariate (right). Labels above the bars indicate *p* values relative to the HFD group. (D) Body surface temperature of DIO mice during low temperature exposure for 4 h (*n* = 5). (E) Representative BAT transmission electron microscope image. Scale, 2 µm. (F) Western blot analysis of BAT HSL in DIO mice. (G) Representative UCP1 immunohistochemical staining in BAT in DIO mice. Scale, 50 µm. (H) Enrichment analysis of Kyoto Encyclopedia of Genes and Genomes (KEGG) pathway of BAT RNA‐Seq differential genes (ASD 150 vs. HFD). (I) *Ucp1* mRNA relative expression in vehicle control and ASD‐treated primary BAT‐derived adipocytes (*n* = 3). (J) Representative phase‐contrast images (left) and oil red o staining (right) of vehicle control or 200 µM ASD‐treated primary BAT‐derived adipocytes. Scale, 50 µm. (K) Fluorescence staining analysis of BODIPY, MitoTracker, and PLIN1 was performed in primary BAT‐derived adipocytes treated with either vehicle control or 200 µM ASD. Purple, PLIN1; red, MitoTracker; green, BODIPY; blue, DAPI for nuclei staining. Scale bar, 20 µm. Statistical significance was defined as *p < 0.05, **p < 0.01, and ***p < 0.001. The significance of the control group versus the model group was expressed as #*p* < 0.05, ##*p* < 0.01, and ###*p* < 0.001. ns, not significant. Significance markers match group colors in the line chart.

To demonstrate that ASD can activate thermogenesis in mice, we measured 24‐h oxygen consumption (VO_2_) and carbon dioxide production (VCO_2_) using an energy metabolism monitoring system. Energy expenditure (EE) and respiratory exchange ratio (RER) were then calculated. In DIO mice, ASD administration elevated VO_2_, VCO_2_, and EE, with nocturnal VO_2_ exceeding diurnal levels (Figures 3A–C and S3A). There was no significant difference in RER, suggesting that the substrate the mice consumed was not biased toward carbohydrates or fat (Figure S3B). The number of activities of the mice during this period was recorded by the light beam sensor, and the feed intake was also documented. Our results revealed that ASD was associated with increased calorie consumption, independent of exercise and diet factors (Figure S3C,D). Generally speaking, mice, as homeothermic animals, do not show significant differences in body temperature under normal temperature conditions. However, in an environment of 4°C, the metabolically active mice were able to maintain their body temperature more effectively. Therefore, we subsequently exposed the mice to a temperature of 4°C in order to assess their ability to adapt to cold conditions. Mice administered with ASD exhibited enhanced thermogenic capacity and higher body surface temperatures compared with those in the HFD group (Figure 3D). We examined the mitochondrial mass of BAT mice through the use of transmission electron microscopy. The consumption of HFD led to a reduction in the quantity of mitochondrial cristae, while ASD helped to alleviate this damage (Figure 3E). Hormone‐sensitive lipase (HSL) is an enzyme that plays a crucial role in regulating the hydrolysis of TGs. ASD has the ability to increase the abundance of HSL protein depending on the dose‐dependent manner (Figure 3F). At the same time, the immunohistochemical staining intensity of UCP1 in BAT was enhanced (Figure 3G). Pathway enrichment analysis conducted by means of transcriptomic sequencing of BAT revealed that, in contrast to HFD mice, the ASD treat mice exhibited a greater number of alterations in genes associated with thermogenesis and oxidative phosphorylation (Figure 3H). ASD significantly upregulated thermogenesis genes in BAT, including *Atp5a*, *Uqcrc2*, *Mtco1*, *Sdhb*, and *Ndufb8* (Figure S3E). In addition, to further clarify the effect of ASD on brown adipocytes, we isolated brown adipose mesenchymal stem cells from BAT; then, induced differentiation into mature brown adipocytes. ASD had no obvious toxic effect on adipocytes (Figure S3F). However, *Ucp1* mRNA expression and protein abundance were increased in mature brown adipocytes in a dose‐dependent manner (Figures 3I and S3G,H). Brown adipocytes exhibit smaller lipid droplets and greater mitochondrial activity, which is a sign that mitochondria are activated and promote fat consumption (Figures 3J,K and S3I). We further examined the effect of ASD on the expression of genes related to fat synthesis, lipolysis and thermogenesis in brown adipocytes. The results showed that ASD promoted the mRNA expression of *Atgl*, *Elovl3*, *Adrb3*, *Dio2*, *Prdm16*, and *Cpt1b* in brown adipocytes (Figure S3J,K). In short, the above results suggest that ASD increases mitochondrial mass in BAT and promotes thermogenesis in mice.

### 2.4 ASD Acts Directly on the Target USP4 in Brown Adipocytes

Studies have reported that proinflammatory factors secreted by macrophages can inhibit adipocyte thermogenesis [31]. It has been reported that ASD is also associated with anti‐inflammatory effects [32, 33]. To confirm the anti‐inflammatory effect of ASD, we first assessed the cytotoxicity of ASD on RAW264.7 cells, observing no discernible toxicity (Figure S4A). Using lipopolysaccharide (LPS) and interferon‐gamma (INFγ) to induce an inflammatory response in RAW264.7 cells, ASD treatment significantly reduced the expression of inflammatory markers *Il1β*, *Il6*, and *Nos2* (Figure S4B). At the same time, we collected the supernatant of RAW264.7 cells and analyzed the concentrations of IL6 and TNFα secreted by the cells. It was observed that ASD significantly decreased the concentrations of released inflammatory factors (Figure S4C). Adipocyte hypoxia‐induced cell death occurs when adipose tissue expansion outpaces vascular oxygen supply. And the clearance of dead adipocytes depends on macrophages. Macrophages phagocytizing dead adipocytes will form characteristic crown‐like structures in the tissue [3]. To observe this crown‐like structures, we detected CD68‐positive cells in BAT, eWAT, and iWAT by immunohistochemical staining. The results indicated that the infiltration of macrophages in BAT and eWAT was significantly reduced after ASD treatment. Interestingly, both eWAT and iWAT are white adipose tissues, but the crown‐like structures in iWAT of mice in the HFD group were far fewer than those in eWAT (Figure S4D). Flow cytometry demonstrated that ASD diminished the recruitment of CD86‐positive cells within BAT (Figure S4E). In a word, ASD treatment can alleviate the infiltration of inflammatory cells in the adipose tissue of mice.

**FIGURE 4:**
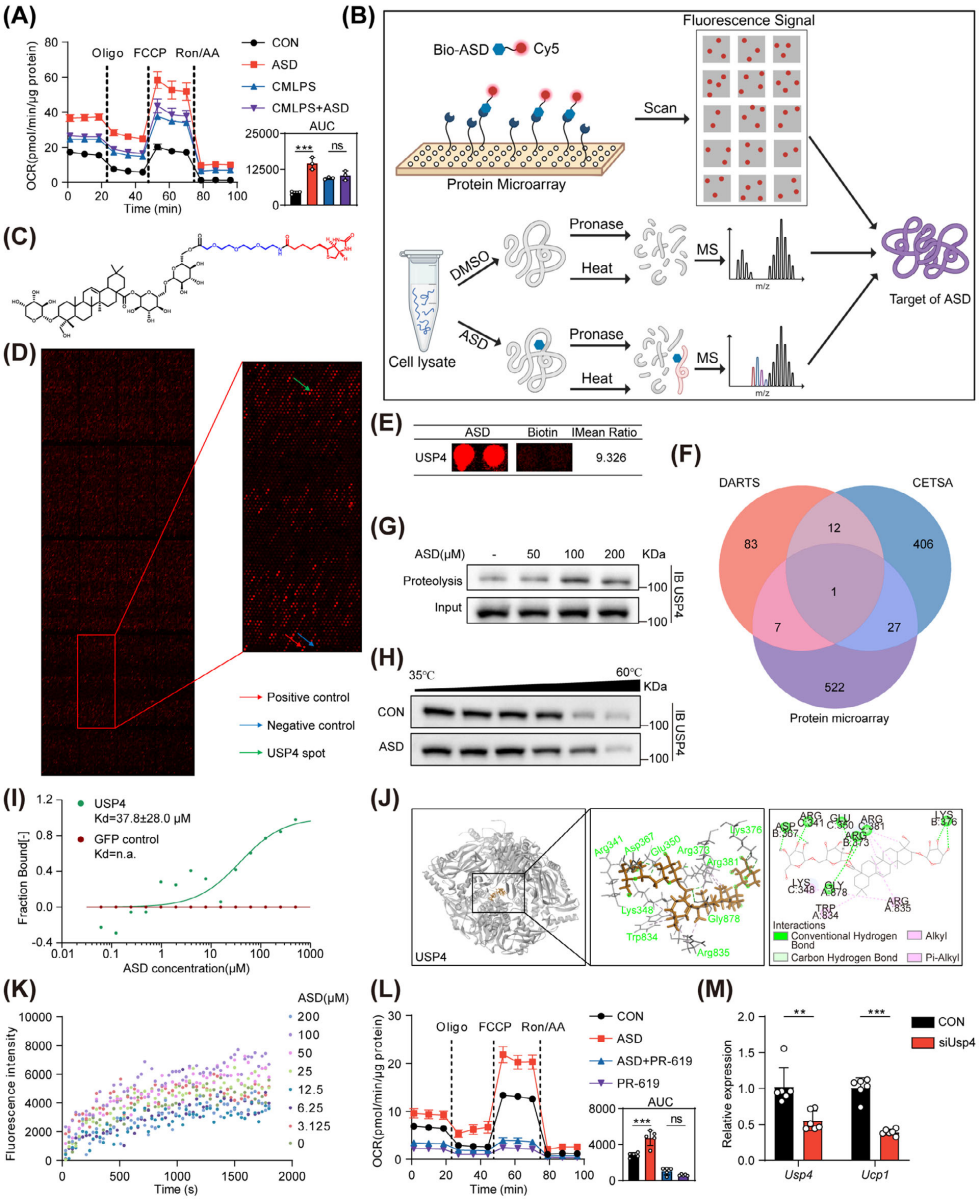
ASD acts directly on the target USP4 on brown adipocytes. (A) LPS + INFγ or LPS + INFγ + ASD were treated for 12 h, and then the conditioned medium was changed and collected for 12 h. They were added 24 h before seahorse testing, and the other two groups were separately added to 200 µM ASD or vehicle control (*n* = 3). (B) Target hook flow chart (created with BioRender.com). Scanning fluorescence analysis after binding of Bio‐ASD and protein array targets. Freshly prepared whole cell lysates were treated with or without ASD, and then digested by protease K or heated at 60°C and analyzed by mass spectrometry. Finally, obtain the intersection of targets. (C) Chemical structure of Bio‐ASD. (D) Representative image of protein array showing positive (red arrow) and negative control (blue arrow) spots, as well as spots for USP4 (green arrow). (E) Enlarged image of Bio‐ASD binding to USP4 points on a protein array. The IMean Ratio is demonstrated in the figure. (The intergroup ratio between Bio‐ASD group and control group was calculated.) (F) The intersection of protein microarray, DARTS, and CETSA. DARTS (G) and CETSA (H) assays confirmed the binding of ASD (200 µM) to USP4 in BAT. (I) Microscale thermophoresis analysis of ASD binding to GFP–USP4 from 293T cell lysates or free GFP. GFP protein was the negative control. (J) Representative images of docking of USP4 and ASD (binding energy = −50.43 ± 1.66 kcal/mol). (K) Effect of ASD on the ability of USP4 to hydrolyze Ub‐Rh110. (L) OCR of primary brown adipocytes treated with vehicle control, 200 µM ASD and/ or PR‐619 (*n* = 5). (M) RT‐qPCR analysis of gene expression in brown adipocytes treated with USP4 siRNA or/and 200 µM ASD (*n* = 6). Statistical significance was defined as *p < 0.05, **p < 0.01, and ***p < 0.001. ns, not significant.

To ascertain whether adipocytes or macrophages are the primary target cells of ASD, LPS + INFγ‐induced conditioned medium (CMLPS) and LPS + INFγ + ASD‐induced conditioned medium (CMLPS + ASD) were collected from RAW264.7 cells. Mature brown adipocytes were cultured with ASD or the conditioned media, followed by a seahorse assay. The results revealed that ASD directly affects brown adipocytes rather than influencing thermogenesis through macrophages (Figure 4A).

In order to discern the precise target of ASD, we employed protein microarray, cellular thermal shift assay (CETSA), and drug affinity responsive target stability (DARTS) experiments to identify the specific proteins that interact with ASD (Figure 4B). First, biotin was connected to ASD (Figure 4C). Next, HuProt proteome microarray was used for target screening (Figure 4D,E). Subsequently, adipose tissue and adipocytes were subjected to DARTS and CETSA assays, respectively, and protein identification was accomplished through liquid chromatography–tandem mass spectrometry (LC–MS/MS). The characteristic peptide of USP4 was identified in both DARTS and CETSA experiments, localized in the 545–559 region of amino acid residues within the ubiquitin‐like 2 (UBL2) domain (Figure S4F). Finally, we combined the results from the three methods and found that USP4 was the only protein consistently identified by all approaches (Figure 4F and Table S3). Therefore, we conclude that the direct target of ASD is USP4.

We also demonstrated by DARTS and CETSA combined Western blot that the addition of ASD to cell lysates reduces the degradation of target USP4 by proteases and temperature (Figure 4G,H). Microscale thermophoresis experiment showed that the affinity constant of ASD binding with USP4 was 37.8 ± 28.0 µM (Figure 4I). We used Discovery Studio to predict that ASD can combine with USP4 (PDB ID: 2Y6E) (Figure 4J). Furthermore, USP4 activity was measured using Ub‐Rh110 as a substrate. Notably, ASD did not markedly influence the capacity of USP4 in catalyzing the degradation of Ub‐Rh110 (Figure 4K). Oxygen consumption rate (OCR) is related to the activity of cellular mitochondria. To verify the effect of USP4 on brown adipocytes OCR, we executed seahorse experiments applying the DUB inhibitor PR‐619. The outcomes indicated that PR‐619 can counteract the effect of ASD in increasing OCR in brown adipocytes (Figure 4L). *Usp4* small interfering RNA (siRNA) was used to interfere with *Usp4* expression. Initially, we validated the *Usp4* siRNA with the best knock‐down capabilities on brown adipocytes (Figure S4G). After interfering *Usp4* expression with siRNA, *Ucp1* mRNA expression was also downregulated (Figure 4M). There was no significant change in *Usp4* mRNA expression in brown adipocytes treated with ASD (Figure S4H). However, USP4 protein abundance was increased in a dose‐dependent manner (Figure S4I). Therefore, we hypothesized that ASD inhibits the degradation of USP4 protein and this was verified by using cycloheximide (CHX) (Figure S4J). These results suggest that ASD directly acts on USP4, and there is a relationship between USP4 and UCP1‐dependent thermogenesis.

### 2.5 USP4 Enhances the UCP1‐Dependent Thermogenesis Pathway by Promoting PPARγ Deubiquitination

**FIGURE 5:**
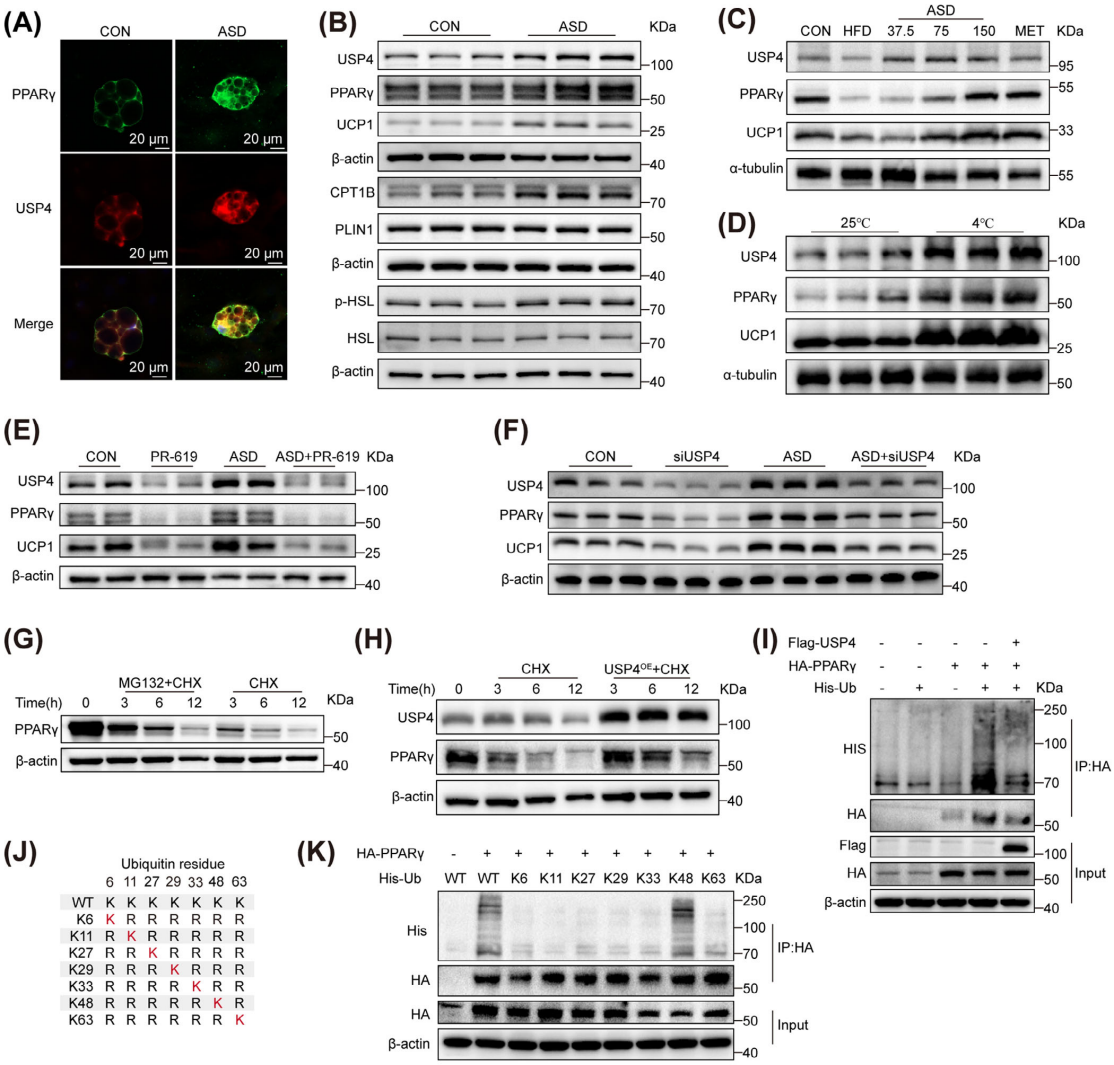
USP4 enhances the UCP1‐dependent thermogenesis pathway by promoting PPARγ deubiquitination. (A) Immunofluorescence analysis shows PPARγ (green) interacting with USP4 (red) in brown adipocytes. Cells were treated with or without ASD (200 µM) for 48 h. Scale, 20 µm. (B) Immunoblots in control and ASD (200 µM)‐treated brown adipocytes. (C) Immunoblots of BAT in DIO mice after ASD treatment. (D) Immunoblots of BAT in C57BL/6 mice after cold exposure or room temperature. (E) Immunoblots of PR‐619 or/and ASD (200 µM)‐treated brown adipocytes. (F) Brown adipocytes were treated with or without ASD (200 µM) immunoblotting after transfection with USP4 siRNA. (G) Brown adipocytes were treated with 25 µg/mL CHX or with CHX and 50 µM MG132. The expression of USP4 was detected by Western blot. (H) 293T cells were transfected with USP4. After 48 h, the cells were treated with 25 µg/mL CHX for different times. The expression of USP4 was detected by Western blot. (I) After the 293T cells overexpressed USP4, PPARγ, and Ub for 48 h, the cells were lysed and processed for immunoprecipitation (IP) with anti‐HA magnetic beads. 50 µM of MG132 was added 4 h before protein collection. (J) Schematic diagram of multipoint mutation of ubiquitin amino acid residues. (K) 293T cells transfected with WT or mutated ubiquitin plasmid to screened for PPARγ ubiquitination types. 50 µM of MG132 was added 4 h before protein collection. Use anti‐HA magnetic beads for IP.

We observed that knockdown of USP4 resulted in inhibition of *Ucp1* mRNA expression. Thus, it is plausible to suggest that USP4 may play a role in regulating the ubiquitination levels of upstream transcription factors of UCP1. Co‐immunoprecipitation (Co‐IP) with anti‐USP4 antibody was performed, followed by LC–MS/MS to identify possible transcription factors. In the results, we found the characteristic peptide of PPARγ (Figure S5A). Subsequently, western blot was performed on the samples obtained after Co‐IP, and the results showed that USP4 interacts with PPARγ (Figure S5B). The interaction between USP4 and PPARγ was further substantiated by immunofluorescence colocalization analysis of brown adipocytes (Figure 5A). Our study observed a positive correlation between the expression of USP4 and the levels of PPARγ and UCP1 in brown adipocytes treated with ASD. Additionally, USP4 levels were directly associated with the abundance of carnitine palmitoyltransferase 1B (CPT1B) protein, which facilitates the uptake of long‐chain fatty acids into mitochondria, without significantly affecting perilipin‐1 (PLIN1) levels. Despite unchanged HSL expression, the abundance of phosphorylated HSL increased in ASD‐treated brown adipocytes (Figures 5B and S5C). PLIN1 regulates lipolysis and TG levels to protect them from the breakdown of HSL [34]. These findings indicate that ASD treatment promotes PPARγ expression, favoring UCP1‐dependent thermogenesis rather than adipocyte differentiation.

In addition, we observed a positive correlation between USP4 and PPARγ in BAT of mice exposed to cold environment and treated with ASD mice (Figures 5C,D and S5D,E). To verify that ASD regulates PPARγ expression through USP4, we used PR‐619 and USP4 siRNA to inhibit or knockdown USP4 in brown adipocytes, which decreased PPARγ abundance and reversed the upregulation of PPARγ induced by ASD (Figures 5E,F and S5F,G). Notably, ASD did not affect PPARγ mRNA levels (Figure S3K), suggesting that PPARγ regulation may occur via the proteasome pathway.

To further confirm whether PPARγ is degraded via the proteasome pathway, we conducted experiments on brown adipocytes using the proteasome inhibitor MG132 and the protein synthesis inhibitor CHX. When protein synthesis is inhibited, MG132 significantly reduce the degradation of PPARγ, indicating that PPARγ degradation is mediated by the proteasome pathway (Figures 5G and S5H). Additionally, overexpression of USP4 in 293T cells potently suppressed PPARγ degradation (Figures 5H and S5I), suggesting that USP4, as the DUB for PPARγ, can reduce its degradation. Subsequent ubiquitination experiments further confirmed the ability of USP4 to deubiquitinate PPARγ (Figure 5I).

To determine whether PPARγ undergoes ubiquitination and to identify the type of ubiquitination modification, we focused on seven lysine residues (K6, K11, K27, K29, K33, K48, and K63) involved in forming Ub chains. We maintained a specific lysine site on the Ub protein while mutating the others with arginine (Figure 5J). Our results revealed that PPARγ ubiquitination primarily occurs via K48‐linked Ub chains (Figure 5K). In summary, we found that the mechanism underlying ASD‐mediated thermogenesis involves USP4‐mediated deubiquitination of PPARγ.

### 2.6 USP4 is Associated with Obesity; the Effect of ASD is Dependent on USP4

**FIGURE 6:**
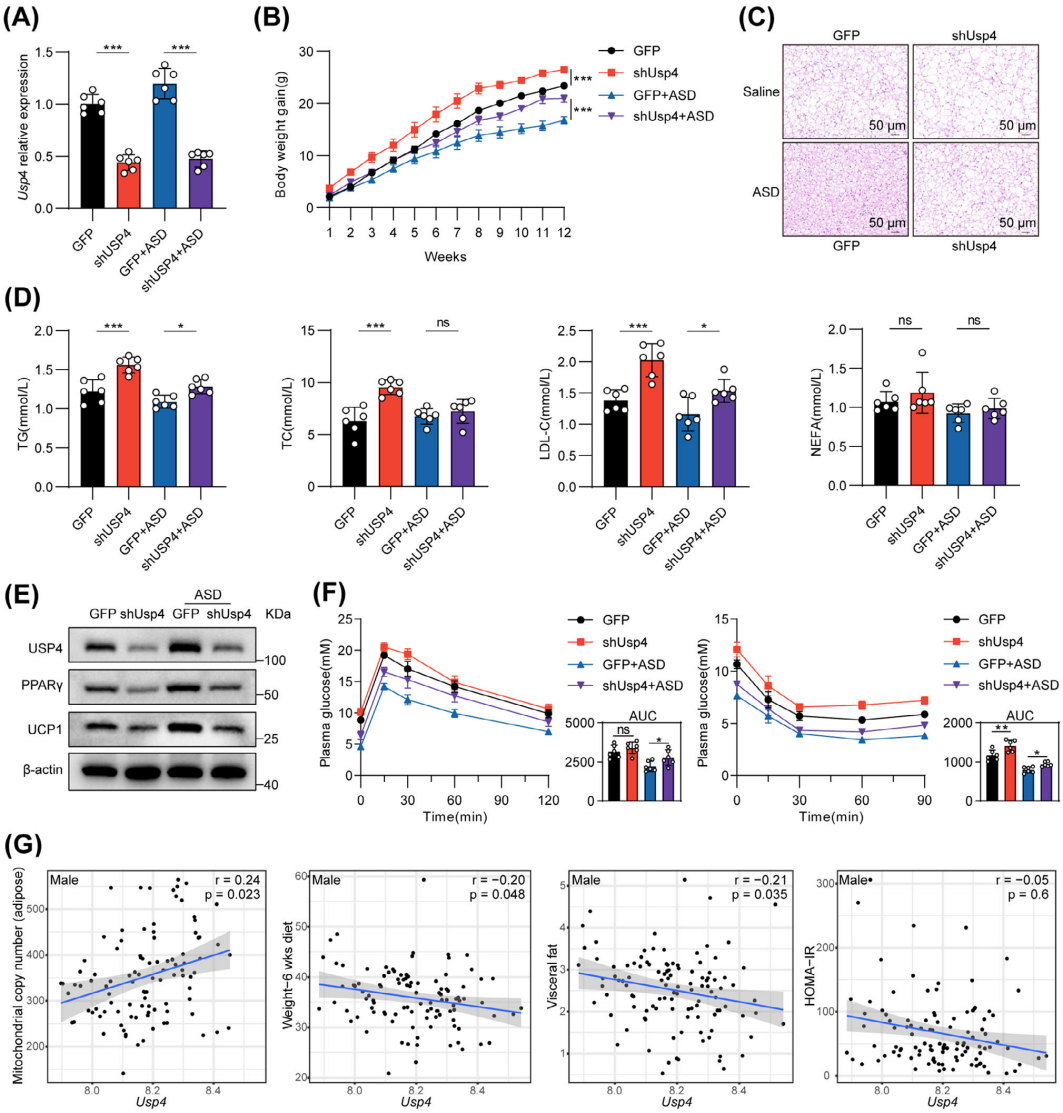
USP4 is associated with obesity, and ASD treatment (150 mg/kg) of diet‐induced obesity in mice is dependent on USP4. (A) Usp4 mRNA expression in BAT (*n* = 6). (B) Weight gain in mice during ASD treatment (*n* = 6). (C) Representative H&E images of BAT. (D) Biochemical detection of mouse blood (*n* = 6). (E) Immunoblotting of UCP1, PPARγ, and USP4 in BAT. (F) GTT and ITT after ASD treatment (*n* = 6). (G) Association of *Usp4* mRNA expression in adipose tissue with mitochondrial copy number, body weight, visceral fat weight, and HOMA‐IR (male). Statistical significance was defined as *p < 0.05, **p < 0.01, and ***p < 0.001. ns, not significant.

To investigate whether the antiobesity effect of ASD on mice is dependent on USP4, we injected lentivirus expressing USP4 short hairpin RNA (Lentivirus‐shUSP4) and then introduced it into the tail vein of UCP1‐cre mice to reduce the expression of *Usp4* in BAT (Figure S6A). The fluorescence signal of GFP was detectable in all four groups of BAT isolated at the termination of drug administration (Figure S6B). Compared with the control Lentivirus‐GFP, injection of Lentivirus‐shUSP4 significantly suppressed USP4 expression in BAT (Figure 6A). ASD reduced weight gain in Lentivirus‐GFP mice, but this effect was completely abolished in Lentivirus‐shUSP4 mice (Figures 6B and S6C). In line with this, the knockdown of USP4 significantly inhibited the reduction in BAT adipocyte area induced by ASD (Figures 6C and S6D). Furthermore, the lipid‐lowering capacity of ASD was markedly impaired following USP4 knockdown (Figure 6D).

ASD administration significantly upregulates the abundance of Ucp1 and PPARγ in BAT of Lentivirus‐GFP mice, but not in BAT of Lentivirus‐shUSP4 mice (Figures 6E and S6E). We previously noted that ASD alleviates insulin resistance in mice, while knockdown of USP4 exacerbates insulin resistance and diminishes the therapeutic efficacy of ASD in mice (Figure 6F). We utilized the Hybrid Mouse Diversity Panel (HMDP) database, developed by Professor Lusis, to perform correlation analysis [35]. Results demonstrated that USP4 expression in adipose tissue positively correlated with mitochondrial DNA copy number in high‐fat/high‐sugar DIO mice. Additionally, a negative correlation was observed between *Usp4* expression and body weight, visceral fat weight, as well as homeostatic model assessment of insulin resistance (HOMA‐IR) levels (Figures 6G and S6F). Together, these results confirm the important role of USP4 in ASD‐mediated thermogenic activation of BAT.

## 3 Discussion

The high‐throughput screening of antitumor drugs using tumor organoids has become a widely employed approach [36]. We employed the UCP1 luciferase reporter gene assay and brown fat organoids to conduct a screening of a natural compound ASD, which can increase UCP1 transcription and protein expression in brown adipocytes through a UCP1‐dependent pathway. In mice, ASD alleviates HFD‐induced obesity and insulin resistance without compromising their activity level. ASD effectively activated heat production in BAT and reduced lipid deposition in white adipose tissue. HFD is associated with mitochondrial damage in BAT [37]. ASD protects mitochondrial function from HFD‐induced damage by increasing Ucp1 levels, enhancing mitochondrial mass, and upregulating oxidative phosphorylation and thermogenic genes in BAT. The above results provide further evidence for the thermogenic properties of small molecule ASD screened by brown fat organoids. There is still a great difference between the physiological and pathological environment of in vitro cultured cells and the body. However, the progress in stem cell and organoid technology has allowed for the development of three‐dimensional cell models that can replicate the histological, molecular, and physiological characteristics of certain human organs. This advancement will play a crucial role in the screening of potential therapeutic compounds for the treatment of various diseases.

ASD is the main active ingredient of *Dipsacus asper Wall*, and the extract of *Dipsacus asper Wall* is mainly used in the treatment of osteoporosis, waist and leg weakness, fractures, and other joint diseases [38, 39]. ASD has been reported to ameliorate metabolic syndrome, relieve skeletal muscle insulin resistance, and treat hepatic steatosis [40, 41, 42]. However, the effects of ASD on adipose tissue thermogenesis have not been previously reported. Our study suggests that the metabolic benefits of ASD in high‐fat dietary patterns are at least partially mediated by ASD‐activated thermogenesis.

Numerous studies have demonstrated the anti‐inflammatory activity of ASD in vitro [33, 43]. In RAW264.7 cells, our research also revealed that ASD could inhibit the inflammatory responses triggered by LPS and INFγ. Although inflammatory cytokine secretion levels were significantly reduced in the ASD‐treated group compared with the model group, the magnitude of reduction was less pronounced than the corresponding mRNA changes. This might be because the process of mRNA translating into proteins is related to the efficiency of the promoter, the availability of the translation initiation complex, and the structure of the mRNA [44]. In addition, the secretory factors in the cell supernatant will increase over time, but the mRNA level can only reflect the gene expression at this moment. The underlying mechanism behind this difference requires further research to be confirmed.

We also noted that ASD could alleviate the inflammatory response in adipose tissue, but the infiltration degree of inflammatory cells in eWAT was much higher than that in iWAT. According to the literature reports, among the two types of white adipose tissues, iWAT belongs to subcutaneous adipose tissue while eWAT belongs to visceral adipose tissue. The number of inflammatory cells in visceral adipose tissue is more abundant than that in subcutaneous adipose tissue, and the infiltration of inflammatory cells in visceral adipose tissue is more severe under the conditions of obesity or insulin resistance [45]. Although some studies have explored the involvement of drugs in adipocyte thermogenesis via macrophages [46], we have not observed such an effect with ASD.

Targeting methods based on affinity of small molecules and proteins, such as chemical crosslinking, photo‐crosslinking, click chemistry, biotin labeling, and other technologies, have been widely used successfully. However, linking small molecules with probes may alter their biological activity or binding specificity [47]. Classical small‐molecule nonlabeling techniques, including DARTS and CETSA, have low sensitivity and low‐abundance target protein signals tend to be masked. This limitation can be optimized in combination with the use of LC–MS/MS. The target of our screening is to obtain the target protein with stable effect and high repeatability. Therefore, we conducted simultaneous biotin‐labeled binding protein microarrays for ASD and target screening for DARTS and CETSA binding LC–MS/MS, the only common target identified across these methods was USP4.

Previous studies have found that PPARγ acts as a transcription factor capable of binding to UCP1 and other thermogenic gene promoters and activating transcription [48, 49, 50]. We found that ASD is functionally regulated primarily by activating the PPARγ–UCP1 axis by targeting USP4. In adipocytes, PPARγ is involved in the regulation of browning of white fat, adaptive thermogenesis production, and adipocyte differentiation [22, 51]. The USP4–PPARγ–UCP1 axis may be the key signaling pathway for brown adipocytes to exert their thermogenic effect. ASD had no significant effect on maker protein PLIN1 in adipocyte differentiation. This may be due to the fact that PPARγ upregulated by ASD is mainly involved in regulating the output of downstream UCP1.

USP4 is a DUB enzyme whose downregulation is associated with insulin resistance, NAFLD, inflammation, and acute injury [30, 52, 53]. In addition, we also found in the HMDP database correlation analysis that USP4 expression decreased in adipose tissue of obese mice. HMDP database has been successfully used to identify metabolism‐related genes [54, 55]. We observed that ASD binds to the UBL2 region of USP4, which interacts with the DUSP and ubiquitin‐like 1 domains and is required for USP4 activation [53]. Although ASD did not affect the catalytic activity of USP4, the protein degradation pathway was inhibited after ASD acted on USP4. The specific mechanism needs to be further studied.

Overall, our study found that ASD‐mediated brown adipose thermogenesis is dependent on upregulation of USP4. Cold exposure also upregulates USP4, which may be a potential target for obesity treatment. Additionally, ASD demonstrates promise for treating insulin resistance and obesity. Structural optimization of ASD as a lead compound could enhance its biological activity while reducing off‐target effects in both animal models and humans. However, our study has several limitations. First, we only focused on the potential therapeutic effect of USP4 in antiobesity through thermogenesis in brown adipocytes. Whether the browning of white adipose tissue also plays a role in this process requires further investigation. Second, existing literature suggests ASD activates the PPARγ pathway in microglia, promoting downstream neurotrophic factor BDNF precursor form (BDNF) expression [56]. Another study indicates that BDNF can activate the UCP1 thermogenic pathway by increasing the noradrenaline content in BAT [57]. This suggests ASD might regulate UCP1 expression in BAT through neural signaling, though the precise mechanism warrants further study. Third, when ASD targets USP4, it does not affect the enzyme activity but is related to its expression. To further explore the effects of ASD targeting USP4, it is necessary to identify the types of epigenetic modifications that USP4 undergoes and generate site mutations or protein segments of USP4 for research. Fourth, it is necessary to determine whether there is a direct interaction between USP4 and PPARγ and then conduct an in vitro ubiquitination reaction to test the inhibitory effect of USP4 on the formation process of multiple Ub chains of PPARγ. Fifth, drug adverse reactions often correlate with off‐target effects. The potential toxicity and side effects of chronic ASD administration require evaluation. Furthermore, ASD may engage additional targets relevant to this study, which merit attention in future research.

## 4 Materials and Methods

### 4.1 Mouse Models

Animal experiments are conducted in accordance with the provisions and general recommendations of the China Laboratory Animal Management Law and approved by the Ministry of Science and Technology. The C57BL/6J (SPF grade) was purchased from Beijing Vital River Company. They were kept in room temperature (22–26°C) and humidity controlled room with light/dark cycle for 12 h. Standard chow diet and sterilized water are given at will. All mice were fed adaptively for 1 week after entering the facility. Male mice aged 8–9 weeks were used in all experiments. In the study of a specific diet, 8–9‐week‐old C57BL/6J was given a high‐fat (60% fat, Research Diet; Cat# D12492) diet for 12 weeks before performing other experiments to produce HFD mice. At the same time as the HFD was started, the HFD mice were treated with ASD by gavage once a day (37.5, 75, and 150 mg/kg), the control and HFD mice were given normal saline. Metformin (200 mg/kg) was given in the MET group.

### 4.2 Brown Adipose Mesenchymal Stem Cells were Isolated and Cultured

C57BL/6J mice aged 5–6 weeks were taken, after anesthesia, BAT was removed from the shoulder area of the back, cut and digested with type 1 collagenase in 37°C water bath for 60 min. Brown adipose mesenchymal stem cells resuspended in medium with DMEM/F12. The cells were filtered with 70 µM cell sieve and transferred to a 37°C, 5% CO_2_ constant temperature cell incubator.

### 4.3 Brown Adipose Mesenchymal Stem Cells Differentiation

Brown adipose mesenchymal stem cells were cultured in DMEM/F12 supplemented with 10% FBS, 5 µg/mL insulin, and 1 nM triiodothyronine (T3) (differentiation medium, DM). Cells were cultured in induction medium with 1 µM dexamethasone, 125 µM indomethacin, 0.5 mM isobutylmethylxanthine, and 100 nM T3 in DM for 2 days. The cells were then cultured in DM until day 8, till they exhibited a fully differentiated phenotype.

### 4.4 Spheroid Formation

Follow the sphere formation method described in the previous article [58]. A total of 50,000 cells were inoculated in 100 µL endothelial growth medium‐2 (PromoCell; Cat# C‐22011) on ultra‐low adherence 96‐well round plate (Corning Life Sciences, USA). The cells were maintained in low serum medium for 2 days until spheroid formation, transferred to low adsorption 24‐well plate (Thermo Scientific, USA), then added to DM and changed every 10 days until detection.

### 4.5 In Vitro Compounds Treatment

A total of 72 compounds related to antiobesity or anti‐inflammation were provided by Selleck Company. Differentiated mature adipocytes were treated with candidate compounds (100 µM) for 24–48 h. For siRNA transfection, Lipofectamine RNAiMAX (Invitrogen; Cat# 13778100) was mixed with siRNA (GenePharma, China) and added to mature fat cells according to the instructions. The sequence of siRNA refers to Table S4. After 24 h, the adherent cells were treated with ASD (200 µM) or vehicle control (0.1% DMSO) for 24–48 h. The inhibitor was preincubated with PR‐619 (5 µM) for 24 h and then treated with ASD (200 µM) for 48 h. 293T cells were transfected with USP4 plasmid and proteins were collected 48 h after transfection with Lipofectamine 3000 (Invitrogen; Cat# L3000001).

### 4.6 RT‐qPCR

Total RNA was extracted from mature adipocytes or mouse BAT using TRIzol reagent (Takara, Japan). Hiscript II reverse transcriptase (Vazyme; Cat# R323) was used. Real‐time fluorescence quantitative PCR system (Thermo Fisher ABI, QuantStudio 5) and SYBRgreen PCR mix (Vazyme; Cat# Q712) were used to analyze the expression levels of specific genes. 18S, tubulin, or β‐actin were selected as reference genes. RT‐qPCR primers are shown in Table S5.

### 4.7 Western Blot

Whole cell lysate was prepared using RIPA lysate buffer containing complete protease inhibitor cocktail. The protein concentration of cell lysate was determined with BCA protein assay reagent. The cell lysate was incubated in SDS‐PAGE sample loading buffer at 98°C for 10 min, separated by 10% SDS‐PAGE, transferred to polyvinylidene difluoride membrane, and incubated with target protein‐specific primary antibody and horseradish peroxidase‐coupled secondary antibody. Proteins were detected using a fully automated GEL imaging analysis system (Bio‐Rad, USA). The sources of the antibodies are shown in Table S6.

### 4.8 Human Protein Microarray

The target protein identification of ASD was performed using a human protein chip at room temperature. Commercial HuProt proteome chips (Bc Bio) were enclosed in PBS containing 3% BSA at room temperature for 1 h. Incubate the protein chip with 10 µM ASD‐linked biotin (Bio‐ASD) in the reaction buffer (PBS containing 1% BSA) at room temperature for 1 h. The microarray was washed three times with PBS and incubated with Cy3‐streptavidin diluted at 1:1000 for 1 h. After rotary drying, the interaction between Bio‐ASD and protein was detected at 635 nm by GenePix 4000B microarray scanner, and biotin was used as a control. GenePixTM Pro v6.0 software was used for data analysis.

### 4.9 BAT USP4 Knockdown Mice

Construct a lentivirus‐based conditional, Cre–Lox‐regulated RNAi vector for Cre‐dependent termination of shRNA expression [59]. UCP1–Cre mice were injected with lentivirus in a tail vein at a dose of 6 × 10^11^ PFU per mouse. After 3 days, the lentivirus‐injected mice were given either normal saline or ASD (150 mg/kg) once a day for 90 days. The lentivirus was supplemented with a dose of 6 × 10^11^ PFU at 45 days. The mice were killed by fasting for 12 h after the last injection of ASD or saline (*n* = 6 per group).

### 4.10 Statistics Analysis

Continuous variables are expressed as mean ± SEM. The normality of the results distribution was evaluated by the or Kolmogorov–Smirnov test. Mann–Whitney (Two groups) or Kruskal–Wallis (multiple groups) nonparametric test was used to analyze correlations for samples that did not satisfy the normal distribution. For results that satisfy a normal distribution; in the case of two groups, the analysis was performed using the Student's *t*‐test. In the case of multiple groups, one‐way analysis of variance was performed using the LSD post hoc test. Each immunoblot and immunofluorescence was performed at least three times. All analyses were performed using SPSS 21.0 software. Visualizations were performed using GraphPad Prism 9 software.

## Author Contributions

Lili Gong, Lihong Liu, and Lang Chen conceived and designed the project. Lang Chen, Donghai Liu, and Yuxi Li performed the experiments. Song Yang, Weihua Jia, Xingbo Wang, and Bing Hu contributed to the experiments. Liang Peng, Honglin Liu, Yuchen Wang, Calvin Pan, and Aldons J. Lusis provided valuable suggestions to improve the project. Lang Chen, Donghai Liu, Lihong Liu, and Lili Gong drafted and/or revised the manuscript. Lili Gong and Lihong Liu supervised the project. All authors have read and approved the final manuscript.

## Ethics Statement

All animal study protocols were approved by the Ethics Committee of China‐Japan Friendship Hospital (no. ZRDWLL230108).

## Conflicts of Interest

The authors declare no conflicts of interest.

## Supporting information




**Figure S1**: Identification of brown fat cell spheres. (A) Fluorescent staining detection of BODIPY and UCP1 in brown fat organoids. Red, UCP1; green, BODIPY; blue, DAPI for nuclei staining. Immunofluorescence image scale bar, 20 µm. H&E staining scale bar, 50 µm.
**Figure S2**: ASD can alleviate insulin resistance in DIO mice. (A) Fasting blood biochemical indices after ASD treatment (*n* = 8). (B) Quantification of eWAT and iWAT and BAT lipid droplet area. (C) Fat mass and lean mass of C57BL/6 mice by MRI scans (*n* = 3). (D) Area under the curve for GTT and ITT (*n* = 8). (E) Fasting insulin and HOMA‐IR (*n* = 5). (F) Representative pancreatic insulin staining and quantitative. Scale bar, 50 µM. Statistical significance was defined as *p < 0.05, **p < 0.01, and ***p < 0.001. The significance of the control group versus the model group was expressed as #*p* < 0.05, ##*p* < 0.01, and ###*p* < 0.001. ns, not significant.
**Figure S3**: ASD activation of thermogenesis was independent of activity levels. (A) Whole‐body VCO_2_ normalized by body weight under basal conditions in DIO mice (*n* = 8). (B) RER in DIO mice (*n* = 8). (C) DIO mice 24 h activity times (*n* = 3). (D) The average food intake of each cage of DIO mice accumulated to the 10th week (*n* = 2). (E) Upregulation of thermogenic genes in BAT RNA‐Seq ASD 150 group compared with HFD group. (F) CCK8 assay of cell viability in ASD‐treated primary brown adipocytes (*n* = 4). (G) Immunoblots of UCP1 in vehicle control and ASD‐treated primary brown adipocytes. (H) Quantification of UCP1 in vehicle control and ASD‐treated primary brown adipocytes. (I) MitoTracker fluorescence intensity in vehicle control and ASD‐treated primary brown adipocytes (*n* = 6). (J) Lipolysis gene mRNA relative expression in vehicle control and 200 µM ASD‐treated primary brown adipocytes (*n* = 3). (K) Thermogenic gene mRNA relative expression in vehicle control and 200 µM ASD‐treated primary brown adipocytes (*n* = 3). Statistical significance was defined as *p < 0.05, **p < 0.01, and ***p < 0.001. The significance of the control group versus the model group was expressed as #*p* < 0.05, ##*p* < 0.01, and ###*p* < 0.001. ns, not significant.
**Figure S4**: ASD reduces inflammatory macrophage infiltration in adipose tissue. (A) CCK8 assay of cell viability in ASD‐treated RAW264.7 cells (*n* = 4). (B) Relative expression of inflammatory gene mRNA in RAW264.7 cells. LPS and INFγ were treated for 12 h, and ASD (200 µM) was added to the other group simultaneously (*n* = 6). (C) The content of inflammatory factors secreted into the medium supernatant of RAW264.7 cells treated in different groups (*n* = 4). (D) Adipose tissue CD68 immunohistochemical staining. Scale, 50 µm. (E) The proportion of CD68 and CD86 positive cells in BAT single cell suspension was detected by flow cytometry. (F) Mass spectrum of USP4 characteristic peptide detected by DARTS and CETSA. (G) Different siRNA interferes with the efficiency of USP4 mRNA expression (*n* = 3). (H) USP4 mRNA expression in brown adipocytes treated with ASD (*n* = 3). (I) Western blot of USP4 in ASD‐treated brown adipocytes. (J) Brown adipocytes were treated with 25 µg/mL CHX or with CHX and 200 µM ASD. The expression of USP4 was detected by Western blot. Statistical significance was defined as *p < 0.05, **p < 0.01, and ***p < 0.001. The significance of the control group versus the model group was expressed as #*p* < 0.05, ##*p* < 0.01, and ###*p* < 0.001. ns, not significant.
**Figure S5**: USP4 can interact with PPARγ. (A) After co‐immunoprecipitation with USP4 antibody, mass spectrum of PPARγ characteristic peptide was detected by LC–MS/MS. (B) The target protein was detected by IP with USP4 antibody. (C) Western blot quantification of ASD in brown adipocytes with or without drug administration. (D) Western blot quantification of BAT protein abundance in DIO mice. (E) Western blot quantification of BAT in 8‐week‐old male C57BL/6J mice exposed to 25°C or 4°C for 16 h. (F) Western blot quantification of PR‐619 or/and ASD‐treated brown adipocytes. (G) Western blot quantification of Brown adipocytes were treated with or without ASD after transfection with USP4 siRNA. (H) PPARγ protein degradation curve. (I) Degradation curve of PPARγ protein during overexpression of USP4. Statistical significance was defined as *p < 0.05, **p < 0.01, and ***p < 0.001. The significance of the control group versus the model group was expressed as #*p* < 0.05, ##*p* < 0.01, and ###*p* < 0.001.
**Figure S6**: Knock down USP4 in mice BAT tissue and induce obesity model. (A) Workflow of ASD administration in BAT‐specific USP4 knockdown mice (created with BioRender.com). (B) Fluorescence imaging was used to analyze the fluorescence intensity of BAT in mice. (C) Representative photos of mice after USP4 knockdown and ASD treatment. (D) Quantification of BAT lipid droplet area. (E)Western blot quantitative analysis of mouse BAT after USP4 knockdown. (F) Association of *Usp4* mRNA expression in adipose tissue with mitochondrial copy number, body weight, visceral fat weight, and HOMA‐IR (female). Statistical significance was defined as *p < 0.05, **p < 0.01, and ***p < 0.001. ns, not significant.
**Table S1**: 72 compounds related to antiobesity or anti‐inflammation.
**Table S2**: Candidate compounds for evaluation of thermogenic potential, related to Figure 1.
**Table S3**: ASD target candidate proteins from protein microarray, CETSA and DARTS intersection relationship analysis, related to Figure 5F.
**Table S4**: Sequences list for siRNA.
**Table S5**: Primer sequences list for qRT‐PCR.
**Table S6**: Antibody resource.

## Data Availability

The datasets used and/or analyzed during the current study are available from the corresponding author on reasonable request. Raw sequencing data for this study can be found in the NCBI SRA database under accession PRJNA1165954: http://www.ncbi.nlm.nih.gov/bioproject/1165954.
