# Supplementary material for: Akebia Saponin D Targeting Ubiquitin Carboxyl‐Terminal Hydrolase 4 Promotes Peroxisome Proliferator‐Activated Receptor Gamma Deubiquitination and Activation of Brown Adipose Tissue Thermogenesis in Obesity
Source: MedComm (2020). 2025 Oct 15;6(11):e70420. doi: 10.1002/mco2.70420 (PMC12521790; doi:10.1002/mco2.70420)
Supplement: Supplementary file 1 — Figure S1: Identification of brown fat cell spheres. (A) Fluorescent staining detection of BODIPY and UCP1 in brown fat organoids. Red, UCP1; green, BODIPY; blue, DAPI for nuclei staining. Immunofluorescence image scale bar, 20 µm. H&E staining scale bar, 50 µm. Figure S2: ASD can alleviate insulin resistance in DIO mice. (A) Fasting blood biochemical indices after ASD treatment (n = 8). (B) Quantification of eWAT and iWAT and BAT lipid droplet area. (C) Fat mass and lean mass of C57BL/6 mice by MRI scans (n = 3). (D) Area under the curve for GTT and ITT (n = 8). (E) Fasting insulin and HOMA‐IR (n = 5). (F) Representative pancreatic insulin staining and quantitative. Scale bar, 50 µM. Statistical significance was defined as *p < 0.05, **p < 0.01, and ***p < 0.001. The significance of the control group versus the model group was expressed as #p < 0.05, ##p < 0.01, and ###p < 0.001. ns, not significant. Figure S3: ASD activation of thermogenesis was independent of activity levels. (A) Whole‐body VCO2 normalized by body weight under basal conditions in DIO mice (n = 8). (B) RER in DIO mice (n = 8). (C) DIO mice 24 h activity times (n = 3). (D) The average food intake of each cage of DIO mice accumulated to the 10th week (n = 2). (E) Upregulation of thermogenic genes in BAT RNA‐Seq ASD 150 group compared with HFD group. (F) CCK8 assay of cell viability in ASD‐treated primary brown adipocytes (n = 4). (G) Immunoblots of UCP1 in vehicle control and ASD‐treated primary brown adipocytes. (H) Quantification of UCP1 in vehicle control and ASD‐treated primary brown adipocytes. (I) MitoTracker fluorescence intensity in vehicle control and ASD‐treated primary brown adipocytes (n = 6). (J) Lipolysis gene mRNA relative expression in vehicle control and 200 µM ASD‐treated primary brown adipocytes (n = 3). (K) Thermogenic gene mRNA relative expression in vehicle control and 200 µM ASD‐treated primary brown adipocytes (n = 3). Statistical significance was defined as *p < 0.05, **p < [file MCO2-6-e70420-s001.docx]

***Supplementary Materials for***

**Akebia Saponin D targeting ubiquitin carboxyl-terminal hydrolase 4 promotes peroxisome proliferator-activated receptor gamma deubiquitination and activation of brown adipose tissue thermogenesis in obesity**

**Authors**

Lang Chen1, Dong-Hai Liu2, Yu-Xi Li3, Song Yang4, Wei-Hua Jia5, Liang Peng1,2, Hong-Lin Liu4, Xing-Bo Wang2, Bing Hu1, Yu-Chen Wang6, Calvin Pan6, Aldons Jake Lusis6, Li-Hong Liu1,2,5*, Li-Li Gong1,2*

**Affiliations**

^1^Institute of Clinical Medical Sciences, China-Japan Friendship Hospital, Capital Medical University, Beijing 100029, China

^2^China-Japan Friendship Hospital (Institute of Clinical Medical Sciences), Chinese Academy of Medical Sciences & Peking Union Medical College, Beijing 100730, China

^3^Beijing Advanced Innovation Center for Soft Matter Science and Engineering, College of Life Science and Technology, Beijing University of Chemical Technology, Beijing 100029, China

^4^China-Japan Friendship Hospital, Beijing, 100029, China

^5^Department of Pharmacy, China-Japan Friendship Hospital, Beijing 100029, China

^6^Division of Cardiology, David Geffen School of Medicine at UCLA, Los Angeles, CA 90095-1679, United States

*Corresponding author. Email: llh-hong@outlook.com, liulihong@zryhyy.com.cn (L.-H.L.); gonglili@126.com, gonglili@zryhyy.com.cn (L.-L.G.)

**Supporting Information Text**

**Luciferase reporter gene experiment**

Adipogenic differentiation was induced by UCP1-luciferase-TDTOMATO WT1 brown adipocytes until day 8. Different compounds were added to the differentiated adipocytes and incubated for 24 h. Chemiluminescence was measured using the Bright-Lite luciferase assay kit (Vazyme, Cat# DD1204).

**Histological analysis**

iWAT, eWAT, and BAT were fixed in 4% (v/v) PFA, paraffin embedded and then sectioned 5 μm. The sections were stained with hematoxylin and eosin (H&E). The size of lipodroplet void was observed under optical microscope and the image was recorded. The H&E staining section data of mice were collected by ImageJ software.

**GTT and ITT**

After fasting for 12 h, the fasting blood glucose of GTT mice was measured, and then 1 g/kg glucose was injected intraperitoneally. The blood glucose was measured at 15, 30, 60 and 120 min, respectively, and the glucose tolerance curve was drawn and the area under the curve (AUC) was calculated. For ITT, after fasting for 4 h, the fasting blood glucose was measured by tail tip blood collection, followed by intraperitoneal injection of 1 IU/kg insulin. The blood glucose was measured at 15, 30, 60 and 90 min. The insulin tolerance curve was drawn and the AUC was calculated.

**Immunohistochemistry**

The 5 μm paraffin sections were dewaxed and hydrated, the antigen was repaired at 100 ℃ for 9 min, washed with PBS, closed with 10% goat serum at room temperature for 20 min, then added with antibody solution, and incubated at 4 ℃ overnight. After rinsed with PBS, 50 μL of secondary antibody was labeled with HRP, incubated at room temperature for 1 h, color was developed by DAB, hematoxylin was re-dyed for 2 min, dehydrated sealing tablets were rinsed with running water, and then photographed under microscope.

**Energy metabolism monitoring system**

The 24 h VO2, VCO2, RER, EE and autonomic activity of mice were measured by small animal Energy Metabolism Monitoring system (Columbus, USA). The mice acclimated to the system for 48 hours before testing began, with free access to food and water. The obtained data were adjusted for body weight for total oxygen consumption VO2. ANCOVA was used to analyze the EE of each group, with body weight as the covariate.

**Cold exposure test**

Mice were placed in an environment of 4 ℃, and the changes of body surface temperature were continuously measured by infrared thermal imager (FLIR-E6390, Estonia) for 30, 60, 90, 150, and 240 min, and the mice were deprived of water.

**RNA sequencing**

BAT samples of high quality RNA were collected, and the purity and concentration of RNA were detected using the NanoDrop 2000 spectrophotometer. To construct the library, Qubit 3.0 fluorescence quantifier was used for preliminary quantification, and the concentration should reach more than 1ng/μL. Subsequently, Qsep400 high throughput analysis system was used to detect the inserted fragments of the library. Then Q-PCR method was used to accurately quantify the effective concentration of the library (effective concentration of the library > 2nM) to ensure the quality of the library. After the library was qualified, PE150 mode sequencing was performed using a high-throughput sequencing platform.

**Viability assay**

Cell viability was measured by cell counting Kit 8. To put it simply, the fat cells were treated with a compound, with 5000 cells/holes in the plate into the 96-well plate. After 12 h, the cells were incubated with 10% CCK8 for 2 h. Cell viability was measured at OD 450 nm by multimode reader (Tecan Spark, USA).

**MitoTracker and Bodipy staining**

Culture medium of differentiated cells was removed from the petri dish. Cells were added to a preheated staining solution containing 250 nM MitoTracker™ red probe (Invitrogen™, Cat# A66442) and 2 μg/mL Bodipy and cultured at 37 ℃ for 30 min. Immunofluorescence staining was performed after fixation (10 min), permeation (10 min), and isolation (10 min). The cells were incubated with PLIN1 at 4 ℃ overnight, then incubated with Alexa Fluor™ 647 at room temperature for 1 h. After encapsulation, confocal microscopy (Zeiss, Germany) was used to capture cell images. Or use multimode reader to detect the fluorescence intensity at excitation wavelength 554 nm/ emission wavelength 576 nm.

**Transmission electron microscope**

Take BAT, cut it into about 1 mm^3^ size on ice, and place it in 2.5% glutaraldehyde phosphate buffer pre-cooled with ice at 4°C for 2 h, and wash it with PBS for 3 times for 10 min each time. Osmium tetroxide (OsO_4_) was fixed for 2 h, dehydrated, impregnated with resin, and polymerized to prepare ultrathin slices of 50-70 nm. The slices were stained with uranyl acetate and lead citrate, observed under transmission electron microscope and photographed.

**Oil red O stain**

Oil red O (0.5% (v/v) dissolved in isopropyl alcohol) and water are mixed at 3:2 and filtered through a 0.45 μm filter. The differentiated adipocytes were washed 3 times with PBS, fixed with 4% (v/v) paraformaldehyde (PFA) for 10 min, and then incubated with oil red O at room temperature for 30 min.

**Seahorse**

The oxidative phosphorylation level of brown adipocytes were measured by XF24 analyzer. Adipocytes were analyzed by OCR in XF24 cell culture microplates, and oligomycin 15 μM, FCCP 10 μM, rotenone and antimycin A 5 μM were added successively.

**Drug Affi nity Responsive Target Stability (DARTS)**

BAT was lysed with M-PER lysis buffer (Thermo Scientific™, Cat# 78501) to obtain protein lysate. After quantification of BCA, the protein concentration was diluted to 5 mg/mL. Add 200 μM ASD or distilled water as vehicle control and store overnight at 4 ℃. Then Streptomyces enzyme or distilled water was precisely incubated at room temperature for 30 min. The enzymolysis products obtained were electrophoretically stained with Coomasil bright blue, and the lysates were detected by quantitative proteomic or western blot.

**Cellular Thermal Shift Assay (CETSA)**

M-PER lysate extracts proteins from brown adipocytes. The cell lysate was treated with 200 μM ASD or distilled water for 4 h. The supernatant was then heated for 3 min at the specified temperature (35-60 ℃) and centrifugally collected for LC-MS/MS or western blot analysis.

**Microscale Thermophoresis**

The interaction between the ASD and the target is measured using the Monolith system. As described in the previous method^56^. USP4 overexpression plasmid with GFP tag was constructed and transfected into 293T cells, and a separate GPF protein was overexpressed as a control. Total protein was extracted from NP40 lysate. The gradient concentration of ASD (500 μmol/L-152.59 nmol/L) was mixed with the protein lysate for detection. The fluorescence intensity varies with time in the temperature gradient. The fluorescence intensity corresponding to the ligand concentration in the selected time period was mapped to a simulated cooperative graph, and the Kd value of the binding affinity constant was calculated automatically by MO. AffinityAnalysis_x86 software.

**Molecular docking of ASD to USP4**

Download the structure file (.sdF) of ASD molecules from the PubChem database. The structure of ASD was optimized by Discovery Studio software to realize 2D / 3D structure conversion, structure pretreatment, energy minimization, etc. Find the USP4 structure data through the PDB database (https://www.rcsb.org/), delete the water molecules and heteroatoms, and hydrogenate. Molecular docking uses the CDocking module in Discovery Studio to set parameters to dock the optimized ASD molecule with the protein target structure.

**Enzyme activity test**

USP4 activity was monitored by fluorescence intensity measurements using a monoubiquitin-Rhodamine substrate (ubiquitin-Rho110, R&D SYSTEMS, Cat# U-555-050). Activity was measured in black polystyrene 96-well plates at 0.5 nM of USP4 at room temperature for 30 minutes. Ubiquitin Rho110 with a substrate concentration of 250 nM was diluted in a series of 2x ratios in a 200 μM DUB buffer [20 mM tris-HCl (pH 8.0), 2 mM CaCl2, 2 mM β-mercaptoethanol]. Immediately after the substrate was added, the fluorescence intensity at the excitation wavelength of 485 nm/ emission wavelength of 535 nm was monitored every 30 seconds with the multimode reader for 30 minutes.

**Co-immunoprecipitation**

Protein A/G Magnetic Beads (sellck, B23201) were divided into two parts, and IgG and USP4 antibodies (abcam, ab245654) were added and incubated for 15 min. The cells were harvested with MPER lysate, the above two samples were added separately, and the rest was used as input. After incubation overnight, loading buffer was added for immunoblotting or IP-MS/MS detection.

**Supplementary Figures and Tables (Figure S1 to Figure S6, Tables S1 to Tables S6).**


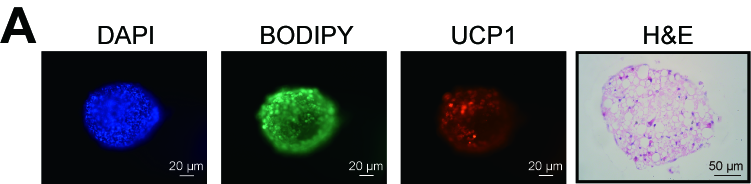


Figure S1. Identification of brown fat cell spheres. (A) Fluorescent staining detection of BODIPY and UCP1 in Brown fat organoids. Red, UCP1; green, BODIPY; blue, DAPI for nuclei staining. Immunofluorescence image scale bar, 20 μm. H&E staining scale bar, 50 μm.


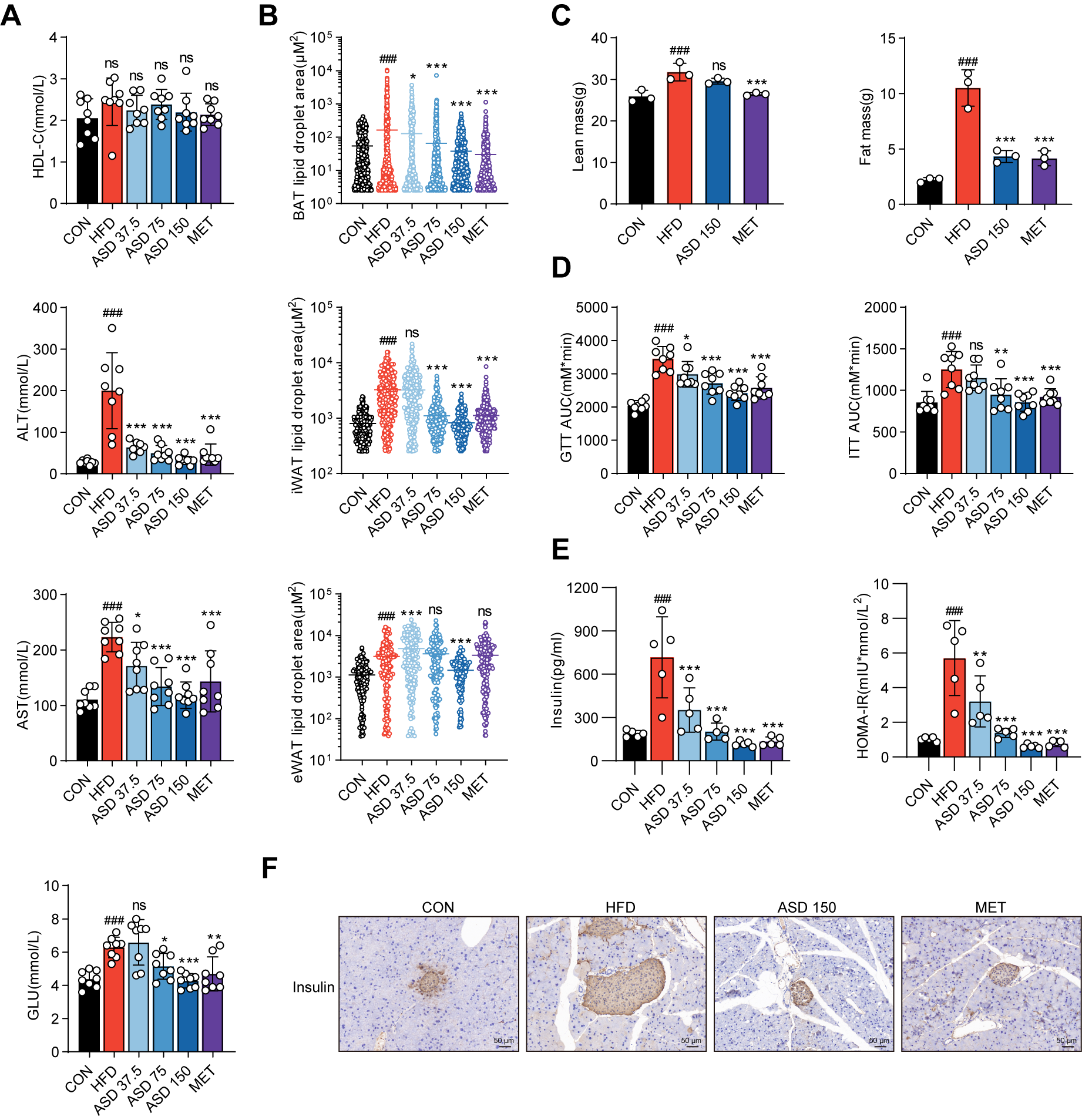


Figure S2. ASD can alleviate insulin resistance in DIO mice. (A) Fasting blood biochemical indices after ASD treatment (n = 8). (B) Quantification of eWAT and iWAT and BAT lipid droplet area. (C) Fat mass and lean mass of C57BL/6 mice by MRI scans (n = 3). (D) Area under the curve for GTT and ITT (n = 8). (E) Fasting insulin and HOMA-IR (n = 5). (F) Representative pancreatic insulin staining and quantitative. Scale bar, 50 μM. Statistical significance was defined as *p < 0.05, **p < 0.01, and ***p < 0.001. The significance of the control group VS the model group was expressed as #p < 0.05, ##p < 0.01, and ###p < 0.001. ns, not significant.


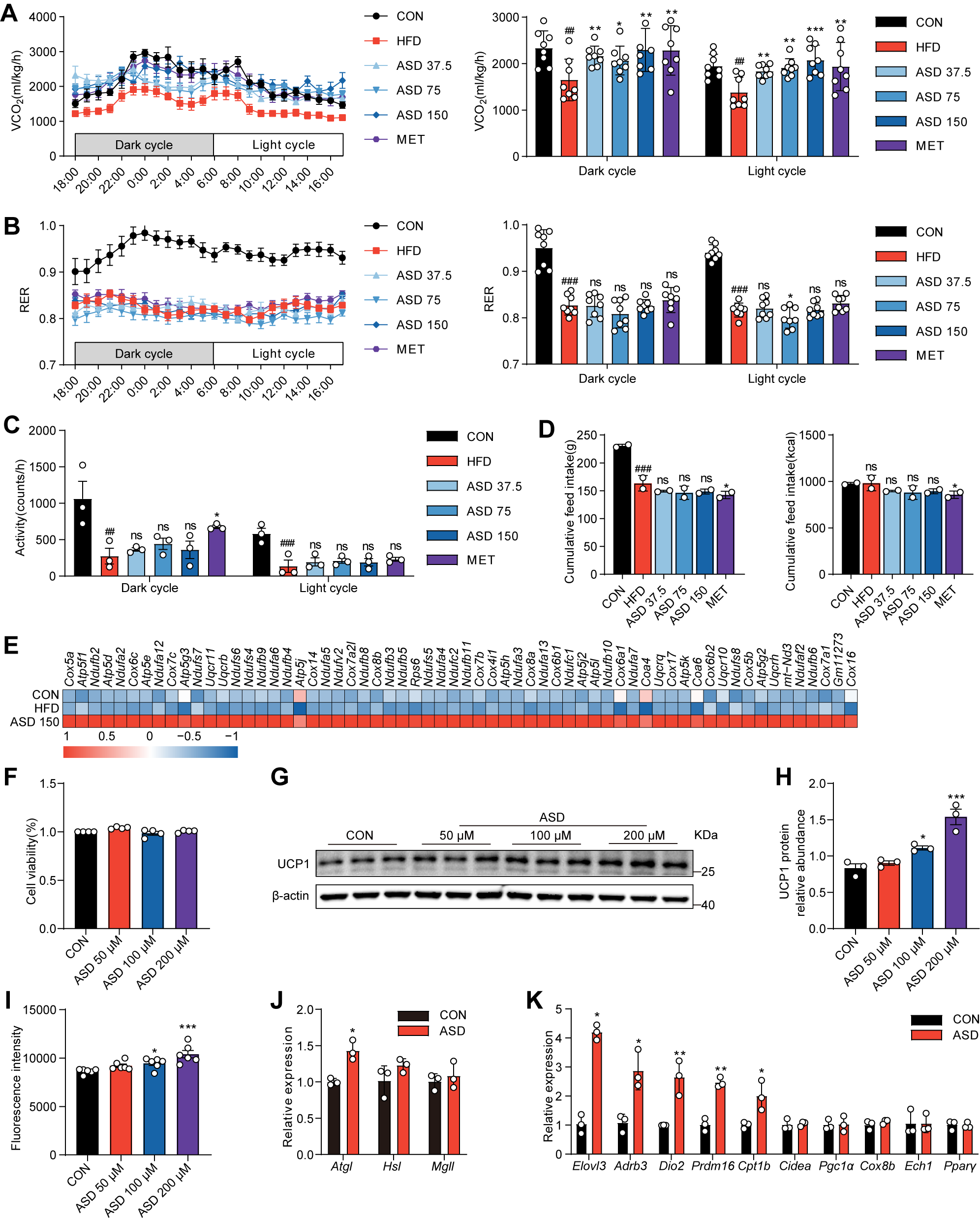


Figure S3. ASD activation of thermogenesis was independent of activity levels. (A) Whole-body VCO_2_ normalized by body weight under basal conditions in DIO mice (n = 8). (B) RER in DIO mice (n = 8). (C) DIO mice 24 h activity times (n = 3). (D) The average food intake of each cage of DIO mice accumulated to the 10th week (n = 2). (E) Upregulation of thermogenic genes in BAT RNA-Seq ASD 150 group compared with HFD group. (F) CCK8 assay of cell viability in ASD treated primary brown adipocytes (n = 4). (G) Immunoblots of UCP1 in vehicle control and ASD treated primary brown adipocytes. (H) Quantification of UCP1 in vehicle control and ASD treated primary brown adipocytes. (I) MitoTracker fluorescence intensity in vehicle control and ASD treated primary brown adipocytes (n = 6). (J) Lipolysis gene mRNA relative expression in vehicle control and 200 μM ASD treated primary brown adipocytes (n = 3). (K) Thermogenic gene mRNA relative expression in vehicle control and 200 μM ASD treated primary brown adipocytes (n = 3). Statistical significance was defined as *p < 0.05, **p < 0.01, and ***p < 0.001. The significance of the control group VS the model group was expressed as #p < 0.05, ##p < 0.01, and ###p < 0.001. ns, not significant.


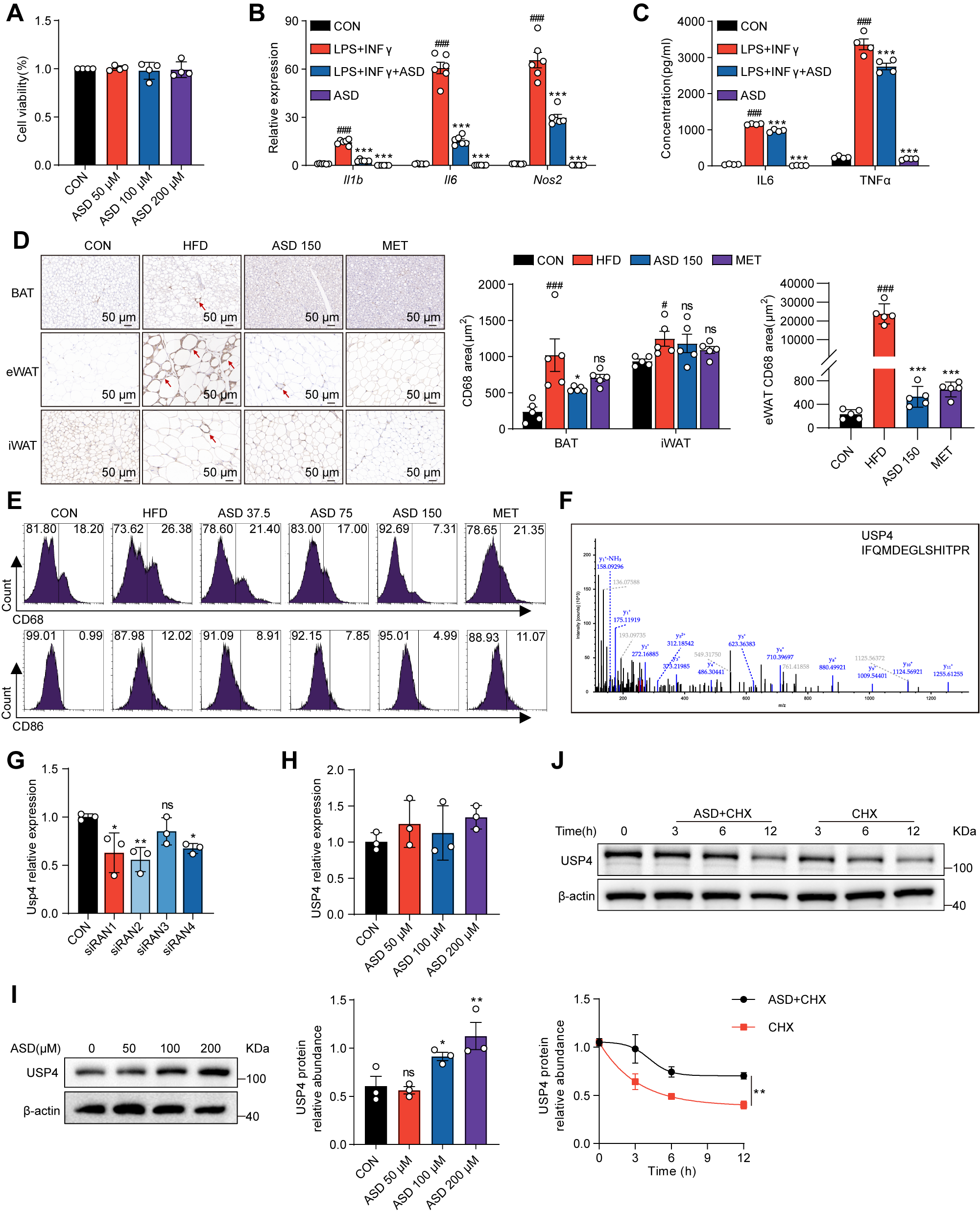


Figure S4. ASD reduces inflammatory macrophage infiltration in adipose tissue. (A) CCK8 assay of cell viability in ASD treated RAW264.7 cells (n = 4). (B) Relative expression of inflammatory gene mRNA in RAW264.7 cells. LPS and INFγ were treated for 12h, and ASD (200 μM) was added to the other group simultaneously (n = 6). (C) The content of inflammatory factors secreted into the medium supernatant of RAW264.7 cells treated in different groups (n = 4). (D) Adipose tissue CD68 immunohistochemical staining. Scale, 50 μm. (E) The proportion of CD68 and CD86 positive cells in BAT single cell suspension was detected by flow cytometry. (F) Mass spectrum of USP4 characteristic peptide detected by DARTS and CETSA. (G) Different siRNA interferes with the efficiency of USP4 mRNA expression (n = 3). (H) USP4 mRNA expression in brown adipocytes treated with ASD (n = 3). (I) Western blot of USP4 in ASD-treated brown adipocytes. (J) Brown adipocytes were treated with 25 μg/mL CHX or with CHX and 200 μM ASD. The expression of USP4 was detected by Western blot. Statistical significance was defined as *p < 0.05, **p < 0.01, and ***p < 0.001. The significance of the control group VS the model group was expressed as #p < 0.05, ##p < 0.01, and ###p < 0.001. ns, not significant.


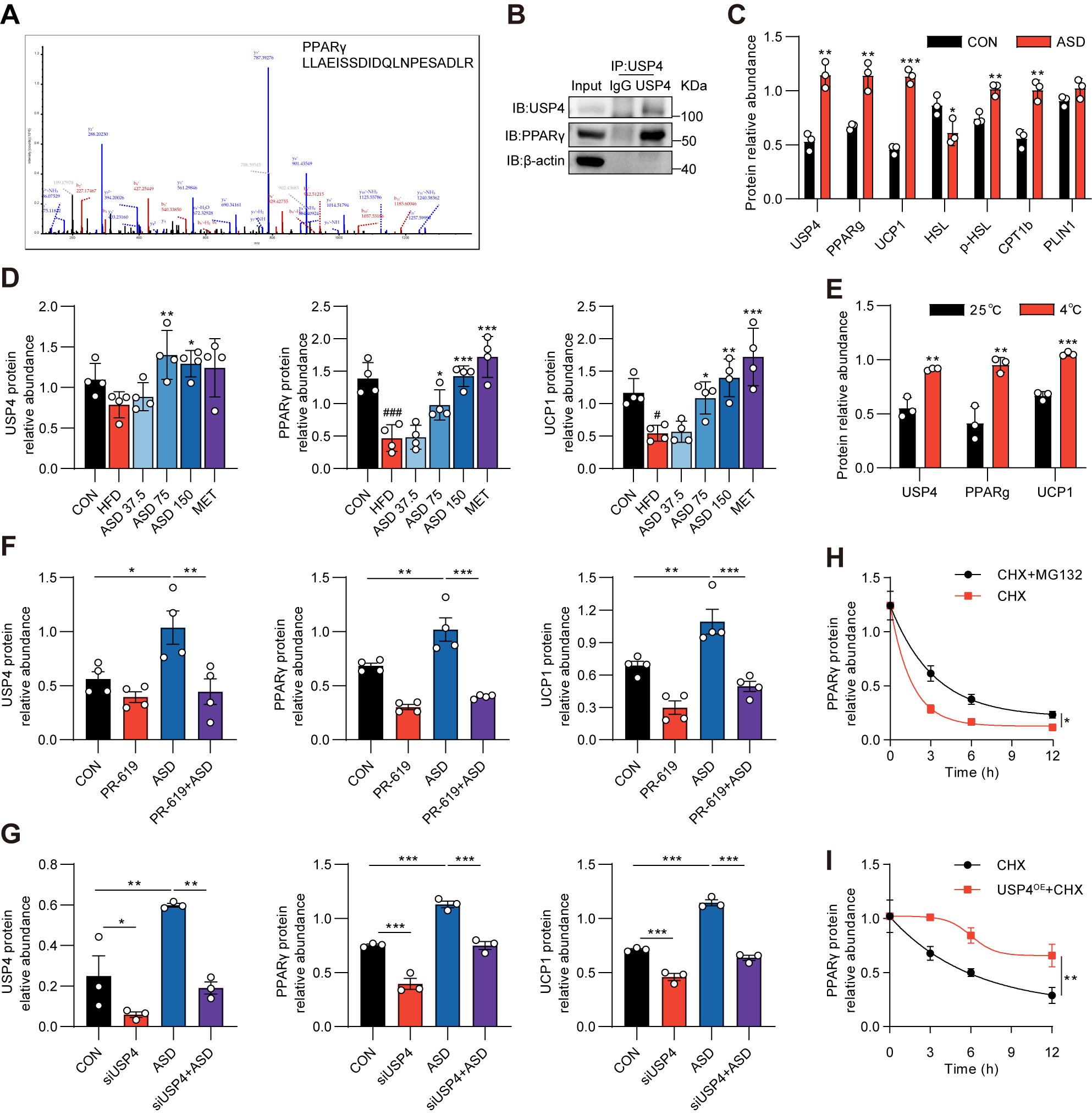


Figure S5. USP4 can interact with PPARγ. (A) After co-immunoprecipitation with USP4 antibody, mass spectrum of PPARγ characteristic peptide was detected by LC-MS/MS. (B) The target protein was detected by IP with USP4 antibody. (C) Western blot quantification of ASD in brown adipocytes with or without drug administration. (D) Western blot quantification of BAT protein abundance in DIO mice. (E) Western blot quantification of BAT in 8-week-old male C57BL/6J mice exposed to 25 ℃ or 4 ℃ for 16 hours. (F) Western blot quantification of PR-619 or/and ASD treated brown adipocytes. (G) Western blot quantification of Brown adipocytes were treated with or without ASD after transfection with USP4 siRNA. (H) PPARγ protein degradation curve. (I) Degradation curve of PPARγ protein during overexpression of USP4. Statistical significance was defined as *p < 0.05, **p < 0.01, and ***p < 0.001. The significance of the control group VS the model group was expressed as #p < 0.05, ##p < 0.01, and ###p < 0.001.


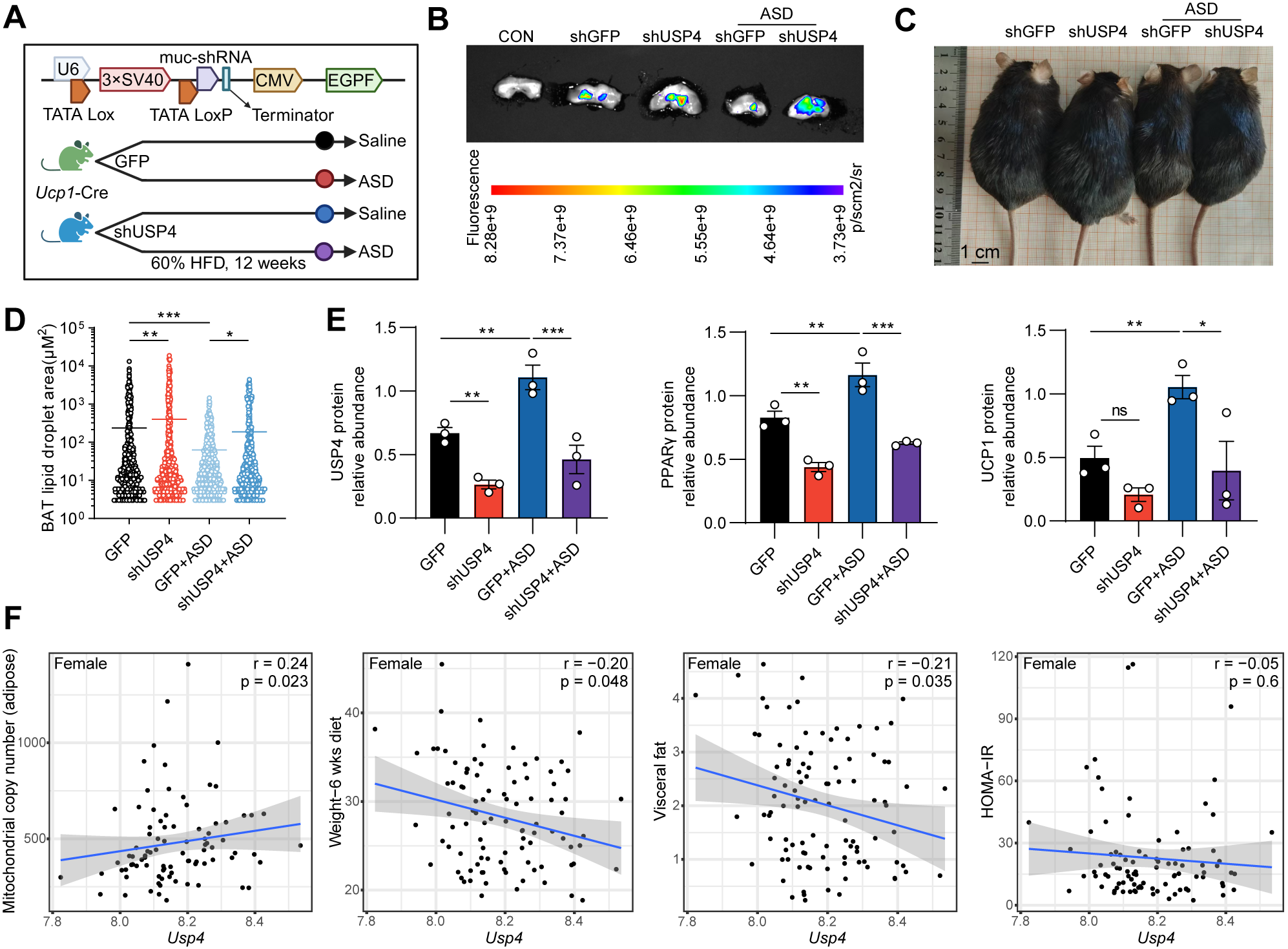


Figure S6. Knock down USP4 in mice BAT tissue and induce obesity model. (A) Workflow of ASD administration in BAT-specific USP4 knockdown mice (Created with BioRender.com). (B) Fluorescence imaging was used to analyze the fluorescence intensity of BAT in mice. (C) Representative photos of mice after USP4 knockdown and ASD treatment. (D) Quantification of BAT lipid droplet area. (E)Western blot quantitative analysis of mouse BAT after USP4 knockdown. (F) Association of USP4 protein expression in adipose tissue with mitochondrial copy number, body weight, visceral fat weight, and HOMA-IR (female). Statistical significance was defined as *p < 0.05, **p < 0.01, and ***p < 0.001. ns, not significant.

Table S1. 72 compounds related to anti-obesity or anti-inflammation.

| **Compound name** | **CAS Number** |
| --- | --- |
| Methoxsalen | 298-81-7 |
| Apigenin (LY 080400) | 520-36-5 |
| Baicalein | 491-67-8 |
| Neohesperidin | 13241-33-3 |
| Tangeretin | 481-53-8 |
| Akebia saponin D | 39524-08-8 |
| Biochanin A | 491-80-5 |
| Asiaticoside | 16830-15-2 |
| Guggulsterone E&Z | 95975-55-6 |
| Schisandrin A | 61281-38-7 |
| Harmine | 442-51-3 |
| Bavachinin | 19879-30-2 |
| Nordihydroguaiaretic acid (NDGA) | 500-38-9 |
| Arctiin | 20362-31-6 |
| Phytol | 150-86-7 |
| Abietic Acid | 514-10-3 |
| Piperlongumine | 20069-09-4 |
| Tanshinone IIA | 568-72-9 |
| Acarbose | 56180-94-0 |
| Genistein (NPI 031L) | 446-72-0 |
| Resveratrol (SRT501) | 501-36-0 |
| Curcumin | 458-37-7 |
| Nicotinamide (NSC 13128) | 98-92-0 |
| Andrographolide | 5508-58-7 |
| Artesunate (WR-256283) | 88495-63-0 |
| Asiatic Acid | 464-92-6 |
| Cryptotanshinone | 35825-57-1 |
| Daidzin | 552-66-9 |
| Kaempferol (NSC 407289) | 520-18-3 |
| (-)-Parthenolide | 20554-84-1 |
| Phloretin (RJC 02792) | 60-82-2 |
| Phlorizin | 60-81-1 |
| Piperine | 94-62-2 |
| Tanshinone I | 568-73-0 |
| Taxifolin (Dihydroquercetin) | 480-18-2 |
| 5-hydroxytryptophan (5-HTP) | 56-69-9 |
| Evodiamine | 518-17-2 |
| Polydatin | 65914-17-2 |
| Quercetin (NSC 9221) | 117-39-5 |
| Naringenin | 480-41-1 |
| Dihydromyricetin | 27200-12-0 |
| Chrysophanic Acid | 481-74-3 |
| Sodium salicylate | 54-21-7 |
| Triptolide (PG490) | 38748-32-2 |
| Cinnamaldehyde | 14371-10-9 |
| Isoferulic Acid | 537-73-5 |
| Gentiopicroside | 20831-76-9 |
| Glabridin | 59870-68-7 |
| Carvacrol | 499-75-2 |
| Mangiferin | 4773-96-0 |
| Euphorbiasteroid | 28649-59-4 |
| Carnosic acid | 3650-09-7 |
| Baohuoside I | 113558-15-9 |
| Iso-Steviol | 27975-19-5 |
| Swertiamarin | 17388-39-5 |
| Acetyl Resveratrol | 42206-94-0 |
| Voglibose | 83480-29-9 |
| Gallic acid | 149-91-7 |
| (+)-Catechin | 154-23-4 |
| Jervine | 469-59-0 |
| Cedrol | 77-53-2 |
| Apocarotenal | 1107-26-2 |
| Oleuropein | 32619-42-4 |
| Ochromycinone (STA-21) | 111540-00-2 |
| Kaempferitrin | 482-38-2 |
| Irigenin | 548-76-5 |
| Chicoric acid | 70831-56-0 |
| Schaftoside | 51938-32-0 |
| Cyanidin-3-O-glucoside chloride | 7084-24-4 |
| Madecassic acid | 18449-41-7 |
| Isoleucine | 73-32-5 |
| Marein | 535-96-6 |

Table S2. Candidate Compounds for Evaluation of Thermogenic Potential, Related to Figure 1.

| **Molecular name** | | ***Ucp1* reporter gene** | | **RT-qPCR** | | **MitoTracker** | |
| --- | --- | --- | --- | --- | --- | --- | --- |
|  |  | **P-value** | **FC** | **P-value** | **FC** | **P-value** | **FC** |
| **Methoxsalen** | 1 | 0.05458 | 1.7429 | 0.01310 | 1.2520 | 0.04446 | 1.3263 |
| **Apigenin** | 2 | 0.05430 | 4.2095 | 0.11374 | 0.8599 | 0.99230 | 0.9994 |
| **Baicalein** | 3 | 0.02380 | 2.8333 | 0.01131 | 1.3330 | 0.00230 | 0.7390 |
| **Neohesperidin** | 4 | 0.10555 | 2.1667 | 0.35176 | 0.9308 | 0.00409 | 1.6661 |
| **Tangeretin** | 5 | 0.00068 | 2.8286 | 0.44836 | 0.9205 | 0.50486 | 1.0395 |
| **Akebia saponin D** | 6 | 0.03395 | 2.7429 | 0.00115 | 2.2366 | 0.01800 | 1.2265 |
| **Biochanin A** | 7 | 0.18515 | 1.7333 | 0.00706 | 0.6954 | 0.61841 | 1.0329 |
| **Asiaticoside** | 8 | 0.00067 | 2.4857 | 0.43737 | 0.9568 | 0.06666 | 1.1280 |
| **Guggulsterone E&Z** | 9 | 0.09635 | 1.6905 | 0.18474 | 0.8839 | 0.05944 | 0.8779 |
| **Schisandrin A** | 10 | 0.00810 | 2.3095 | 0.00156 | 0.6054 | 0.37811 | 1.0551 |
| **Harmine** | 11 | 0.07094 | 0.5667 | 0.00016 | 2.3962 | 0.00011 | 1.5648 |
| **Bavachinin** | 12 | 0.41891 | 1.0286 | 0.00487 | 5.4677 | 0.60508 | 0.9672 |
| **Nordihydroguaiaretic acid** | 13 | 0.91330 | 0.9714 | 0.00365 | 2.8835 | 0.04037 | 0.8639 |
| **Arctiin** | 14 | 0.00208 | 2.3429 | 0.57360 | 0.9564 | 0.37223 | 0.9366 |
| **Phytol** | 15 | 0.21976 | 1.3714 | 0.01006 | 1.3312 | 0.35728 | 0.9419 |
| **Abietic Acid** | 16 | 0.01021 | 2.3429 | 0.00044 | 1.6303 | 0.09040 | 1.1127 |
| **Piperlongumine** | 17 | 0.00219 | 0.0714 | 0.09953 | 1.1884 | 0.06892 | 1.2277 |
| **Tanshinone IIA** | 18 | 0.18963 | 2.3143 | 0.00348 | 1.4661 | 0.06155 | 1.2667 |

Table S3. ASD target candidate proteins from Protein microarray, CETSA and DARTS intersection relationship analysis, Related to Figure 5. F.

| **Protein microarray and CETSA** | **CETSA and DARTS** | **Protein microarray and DARTS** |
| --- | --- | --- |
| USP4 | USP4 | USP4 |
| ALOX5 | TRADD | BTF3 |
| UGP2 | MAP2K3 | IGKC |
| PGM3 | ARAP1 | RABGGTB |
| TPP2 | CUL1 | PRDX4 |
| EIF2B1 | NRAS | RNH1 |
| ADI1 | ACTR10 | FARSB |
| CBWD1 | TRAPPC11 | EIF2B3 |
| CFAP36 | PLCG2 |  |
| UBE2R2 | ERP44 |  |
| TSR1 | WASHC5 |  |
| THUMPD1 | SNRNP200 |  |
| STAT3 | AUH |  |
| EXOC5 |  |  |
| RTN4 |  |  |
| BAG6 |  |  |
| MYL6 |  |  |
| PSME2 |  |  |
| EEF1D |  |  |
| CCT8 |  |  |
| HEATR3 |  |  |
| CDC42 |  |  |
| ZW10 |  |  |
| CAST |  |  |
| UNC45A |  |  |
| RUFY3 |  |  |
| SUGT1 |  |  |
| TTC1 |  |  |

Table S4 Sequences list for siRNA.

| ***Usp4*(mouse)** | **Sequence** | |
| --- | --- | --- |
|  | **sense（5'-3'）** | **antisense（5'-3'）** |
| **siRNA1** | GCGUAAAGAAGAAGCCUUATT | UAAGGCUUCUUCUUUACGCTT |
| **siRNA2** | CCAAAUGGAUGAAGGUUUATT | UAAACCUUCAUCCAUUUGGTT |
| **siRNA3** | GUUCACCUCAAGCGUUUCUTT | AGAAACGCUUGAGGUGAACTT |
| **siRNA4** | GCCUUAUGUUUAUGACCUATT | UAGGUCAUAAACAUAAGGCTT |

Table S5 Primer sequences list for qRT-PCR.

| **Genes(mouse)** | **Primer sequence** |
| --- | --- |
| ***18s*** | F: CGCCGCTAGAGGTGAAATTCT |
|  | R: CATTCTTGGCAAATGCTTTCG |
| ***β-actin*** | F: GTGACGTTGACATCCGTAAAGA |
|  | R: GCCGGACTCATCGTACTCC |
| ***Ucp1*** | F: CAGCCTACAGAGGTCGTGAA |
|  | R: GCATTGTAGGTCCCCGTGTA |
| ***Pparγ*** | F: GCTGACCCAATGGTTGCTGA |
|  | R: AGCCTGATGCTTTATCCCCAC |
| ***Atgl*** | F: TCCGGTGGATGAAAGAGCAG |
|  | R: TCCCCCAGTGAGAGGTTGTT |
| ***Hsl*** | F: GCTATGTGGCTTCTAACCGCA |
|  | R: CAGCCTTTGTGTAGCGTGAC |
| ***Mgll*** | F: AGGATGGTGGTGTCGGACTT |
|  | R: TCGGGGTAGTCCTTCTGGAT |
| ***Elovl3*** | F: TTCTCACGCGGGTTAAAAATGG |
|  | R: GAGCAACAGATAGACGACCAC |
| ***Adrb3*** | F: GGCCCTCTCTAGTTCCCAG |
|  | R: TAGCCATCAAACCTGTTGAGC |
| ***Dio2*** | F: CCACCTGACCACCTTTCACTA |
|  | R: GCACATCGGTCCTCTTGGTT |
| ***Prdm16*** | F: CCCCACATTCCGCTGTGAT |
|  | R: CTCGCAATCCTTGCACTCA |
| ***Cpt1b*** | F: GCACACCAGGCAGTAGCTTT |
|  | R: CAGGAGTTGATTCCAGACAGGTA |
| ***Cidea*** | F: CTTCCTCGGCTGTCTCAATGT |
|  | R: GGAACTGTCCCGTCATCTGTG |
| ***Pgc1α*** | F: TATGGAGTGACATAGAGTGTGCT |
|  | R: CCACTTCAATCCACCCAGAAAG |
| ***Cox8b*** | F: TGTGGGGATCTCAGCCATAGT |
|  | R: AGTGGGCTAAGACCCATCCTG |
| ***Ech1*** | F: GCTACCGCGATGACAGTTTC |
|  | R: TCAGAGATCGAAGGCTGATGTT |
| ***Il1β*** | F: GAAATGCCACCTTTTGACAGTG |
|  | R: TGGATGCTCTCATCAGGACAG |
| ***Il6*** | F: CTGCAAGAGACTTCCATCCAG |
|  | R: AGTGGTATAGACAGGTCTGTTGG |
| ***Nos2*** | F: GTTCTCAGCCCAACAATACAAGA |
|  | R: GTGGACGGGTCGATGTCAC |
| ***Tnfα*** | F: CCTGTAGCCCACGTCGTAG |
|  | R: GGGAGTAGACAAGGTACAACCC |

Table S6. Antibody Resource.

| **Antibody** | **Source** | **Identifier** |
| --- | --- | --- |
| Rabbit anti-USP4 | Abcam | Cat# ab245654 |
| Rabbit anti-UCP1 | Cell Signaling Technology | Cat# 72298; RRID: AB_2936479 |
| Rabbit anti- Perilipin-1 (PLIN1) | Cell Signaling Technology | Cat# 9349; RRID: AB_10829911 |
| Rabbit anti-p-HSL (Ser565) | Cell Signaling Technology | Cat# 4137; RRID: AB_2135498 |
| Rabbit anti-PPARγ | Proteintech | Cat# 16643-1-AP; RRID: AB_10596794 |
| Rabbit anti-HSL | Proteintech | Cat# 17333-1-AP; RRID: AB_2878386 |
| Rabbit anti-CPT1B | Proteintech | Cat# 22170-1-AP; RRID: AB_2713959 |
| Rabbit anti-Flag Tag | Proteintech | Cat# 20543-1-AP; RRID: AB_11232216 |
| Rabbit anti-HA Tag | Proteintech | Cat# 51064-2-AP; RRID: AB_11042321 |
| Mouse anti-β-actin | Proteintech | Cat# 66009-1-Ig; RRID: AB_2687938 |
| Mouse anti-α-tubulin | Proteintech | Cat# 66031-1-Ig; RRID: AB_11042766 |
| Mouse anti-His Tag | Proteintech | Cat# 66005-1-Ig; RRID: AB_11232599 |
| Alexa Fluor™ 647 donkey anti-rabbit lgG(H+L) | Thermo Fisher Scientific | Cat# A-31573; RRID: AB_2536183 |
| Rabbit Anti-CD68 | ServiceBio | Cat# GB113109; RRID:AB_2935658 |
| APC anti-mouse CD34 | BioLegend | Cat# 152215; RRID: AB_2910310 |
| PE anti-mouse CD68 | BioLegend | Cat# 137014; RRID: AB_10612937 |
| Brilliant Violet 421(TM) anti-mouse CD86 | BioLegend | Cat# 105031; RRID: AB_2650895 |
